# Arsenolipids are not uniformly distributed within two brown macroalgal species *Saccharina latissima* and *Alaria esculenta*

**DOI:** 10.1007/s00216-019-01907-x

**Published:** 2019-05-31

**Authors:** Ásta H. Pétursdóttir, Jonathan Blagden, Karl Gunnarsson, Andrea Raab, Dagmar B. Stengel, Jörg Feldmann, Helga Gunnlaugsdóttir

**Affiliations:** 1 Matís, Research and Innovation, Vínlandsleið 12, 113 Reykjavík, Iceland; 2 Trace Element Speciation Laboratory Aberdeen, University of Aberdeen, Meston Walk, Aberdeen, AB24 3UE UK; 3 Marine & Freshwater Research Institute, Skúlagata 4, 101 Reykjavík, Iceland; 4 Botany and Plant Science, School of Natural Sciences, and, Ryan Institute for Environmental, Marine and Energy Research, National University of Ireland Galway, Galway, Ireland

**Keywords:** Mass spectrometry/ICP-MS, Accurate mass, Identification, Quantification, Seaweed, Arsenic speciation

## Abstract

Brown macroalgae *Saccharina latissima* (30–40 individuals) and *Alaria esculenta* (15–20 individuals) were collected from natural populations in winter in Iceland. The algal thalli were sectioned into different parts (e.g. holdfast, stipe, old frond, young frond and sori-containing frond sections) that differed in age and biological function. The work elucidated that arsenic (As) was not uniformly distributed within the two brown macroalgal species, with lower levels of total As were found in the stipe/midrib compared to other thallus parts. The arsenosugars mirrored the total arsenic in the seaweed mainly due to AsSugSO_3_ being the most abundant As species. However, arsenic speciation using parallel HPLC-ICP-MS/ESI-MS elucidated that the arsenic-containing lipids (AsL) had a different distribution where the arsenosugarphospholipids (AsPL) differed by approximately a factor of 4 between the sections containing the lowest and highest concentrations of AsPLs. When placing the sections in order of metabolic activity and an estimate of tissue age, there appeared to be a relationship between the activity and AsPLs, with lower levels of AsPLs in oldest parts. This is the first time such a relationship has been shown for AsLs. Hence, by applying sophisticated analytical techniques, it was possible to gain a deeper understanding of arsenolipids in seaweed.

**Electronic supplementary material:**

The online version of this article (10.1007/s00216-019-01907-x) contains supplementary material, which is available to authorized users.

## Introduction

Arsenic is a ubiquitous element in the environment and is found as over 100 naturally occurring arsenic species [1, 2]. These species exist as both organic and inorganic forms of arsenic and their toxicity is species dependent [3]. There has been significant interest in the inorganic arsenic (iAs, arsenite and arsenate) since it is a known carcinogen [4]. With more information and better analytical techniques to determine iAs, the current legislative focus is shifting from total arsenic (totAs) to iAs [5] due to very different toxicological properties of arsenic species. Organic arsenic has generally been considered less toxic and not a major concern with regard to human health. However, in recent years, light has been shed on the high toxicity of arsenolipids (AsLs) [6]. AsLs can be grouped based on their structure. The main groups of AsLs are arseno-hydrocarbons (AsHCs), arseno-fatty acids (AsFAs) and mono- and di-acyl arsenosugarphospholipids (AsPLs also referred to as AsSugPLs) [7–9]. The phospholipids containing As are more diverse than only the AsPLs (i.e. AsSugPLs) and include arsenic-containing phosphatidylcholines (AsPCs) and arsenic-containing phosphatidylethanolamines (AsPEs) [10, 11]. The arseno-fatty alcohols (TMAsFOHs) are the only AsL group comprising a trimethyl arsenio moiety instead of a dimethyl arsenio moiety [12]. Yu et al. [10] suggest that due to the diversity, i.e. that phosphatidyl lipids can contain both arsenic bound to fatty acids and sugars, these AsLs are formed after the biosynthesis of the arsenic-containing moiety. However, how these species are formed is still unclear.

The AsLs appear to be an emerging threat in food safety, particularly the AsHCs. The AsHCs have shown similar cytotoxicity to iAs [13] although the mode of action of iAs and AsHCs may be different [13, 14]. AsLs pass the in vitro intestinal barrier model (Caco-2) as well as the blood–brain barrier (BBB) of fruit flies (in vivo) and porcine (in vitro) [6, 15, 16]. AsHC360 possesses very high toxic potential where it is five times more cytotoxic than iAs. AsHC332 and AsHC360 disrupt the in vitro BBB and are effective permeability enhancers and might therefore facilitate the transfer of other foodborne toxicants into the brain [6]. AsLs have also been found in human breast milk [17]. Seaweed can contain high amounts of AsHCs, where the toxic AsHC360 has been shown to be the dominant AsL in brown filamentous algae such as Ectocarpus spp., *Pylaiella littoralis* (L.) Kjellm. and Elachista sp. [18]. The edible Hijiki (*Sargassum fusiforme* (Harvey) Setchell), Kombu (*Saccharina japonica* (Aresch.) C.E. Lane, C. Mayes, Druehl & G.W. Saunders) and dulse (*Palmaria palmata* (L.) F. Weber & D. Mohr) have significant proportions of AsHCs of the total AsLs (20–50%) [10, 19, 20]. Due to high amounts of iAs in Hijiki, its consumption has been discouraged for a number of years [5]. The cultivation of *Saccharina latissima* and *Alaria esculenta* has received significant attention as species for human consumption in Europe due to their potentially high biomass yields and nutrient content [21–23]. When considering human consumption, the blades are of highest interest since, e.g. when multiple partial harvest of cultivated seaweed is employed, the holdfast and stipe are left on the ropes [22]. Here we investigate if AsLs are uniformly distributed within the edible brown algal (kelp) species *Saccharina latissima* and *Alaria esculenta* which may provide clues to where these species are formed or stored.

## Materials and methods

### Chemicals and materials

All materials used were of analytical grade or better unless otherwise stated. MilliQ (18 MΩ cm) water was used throughout. A 1000 mgL^−1^ As stock solution (as H_3_AsO_4_) was purchased from Peak Performance (CPI International, USA) as were Te (1000 mgL^−1^) and In (1000 mgL^−1^) standards for use as internal standards, for calibration of arsenic species. Dimethylarsinic acid disodium salt (100%) supplied by Argus-Chemicals (Italy) was dissolved in MilliQ water and diluted as required. Disodium methyl arsonate hexahydrate (99.5%) was supplied by Chem-Service (USA); disodium hydrogen arsenate heptahydrate (As^V^), sodium arsenite (As^III^) and ammonium carbonate were purchased from BDH (UK). Chemicals used for extractions were purchased from Sigma-Aldrich: MeOH (> 99.9%), DCM (> 99.8%), hexane (> 97%). Hydrogen peroxide (puriss p.a., > 30%) was also purchased from Sigma-Aldrich. Two nitric acids were used: trace select (> 69%) was purchased from Fluka (UK) and puriss p.a. (> 65%) was purchased from Sigma-Aldrich; the puriss p.a. acid was used throughout unless otherwise stated.

### Sample collection and preparation

**Fig. 1:**
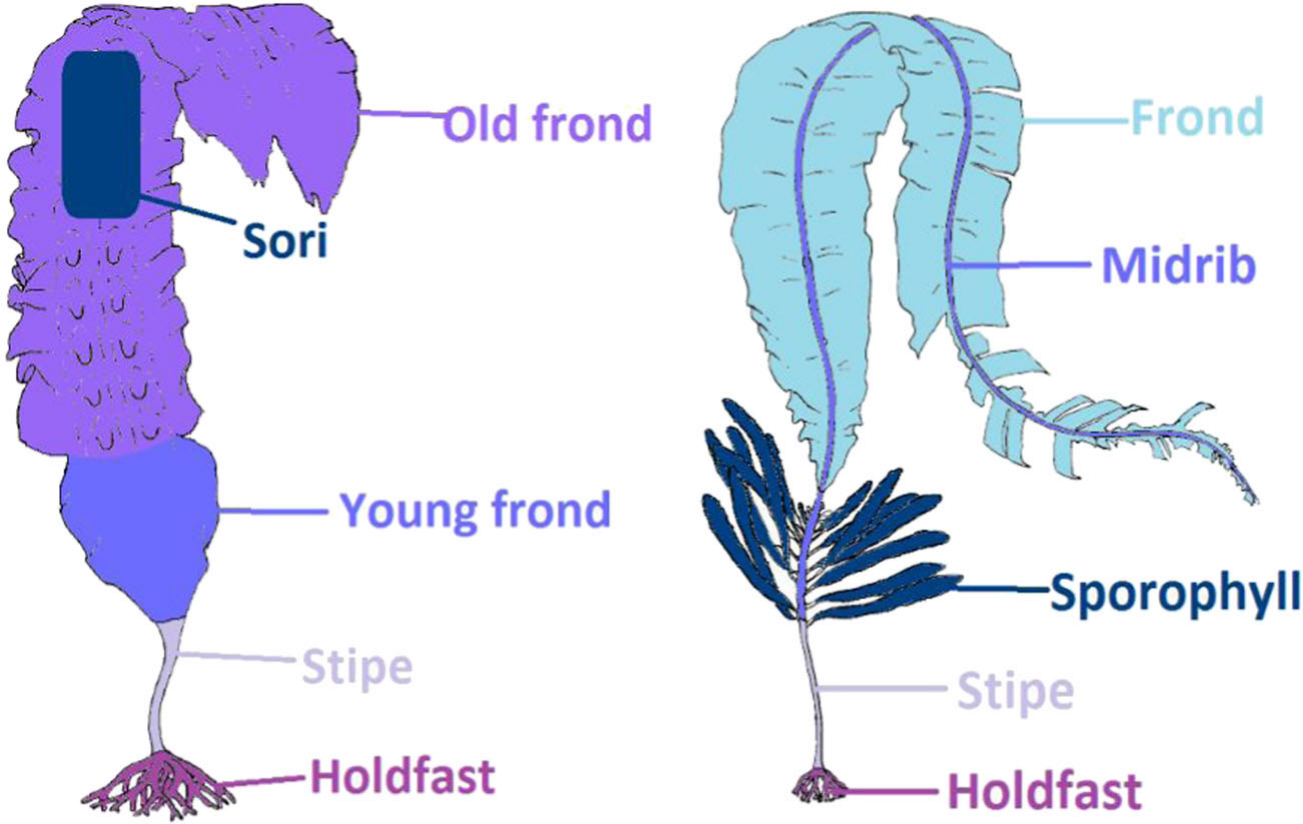
Diagram of sections cut from **a***Saccharina latissima* and **b***Alaria esculenta*. Figures adapted from [24]

Samples of seaweed were collected from the northern coast of the Reykjanes peninsula in the southwest region of Iceland (January 2017). Approximately 1.5 kg of *Saccharina latissima* (L.) C.E. Lane, C. Mayes, Druehl & G.W. Saunders (30–40 individuals) and 500 g of *Alaria esculenta* (L.) Grev. (15–20 individuals), both Phaeophyceae (Ochrophyta), were collected ensuring that the holdfast was taken as intact as possible. A lower mass of *A. esculenta* was collected as the individual samples were smaller than *S. latissima*, and *A. esculenta* grows at a lower shore level, making it less accessible for sampling. The seaweed was transported back to the lab in plastic bags and stored overnight in a fridge at 4 °C. The seaweed material was then manually cleaned of epiphytes, epizoa and detritus with the aid of tap water. The cleaned samples were then rinsed with MilliQ water and cut into sections which were pooled together for all samples of each species, Fig. 1, and subsequently freeze-dried and ground to a fine powder.

As in other kelps, meristematic growth of *S. latissima* occurs at the base of the frond, with the youngest region closest to the stipe. The old frond may be damaged by wave action and other environmental stressors, and decay can be particularly pronounced during summer when growth is slowest. *S. latissima* samples were divided into holdfast, stipe, young frond, sori-containing frond sections and old fronds. The distinction between ‘old’ frond and ‘young’ frond was determined as the point where the frond narrowed; this was apparent on all samples and is purported to be caused by the reduced growth rate over the summer period. Sori of *S. latissima* were identified as visibly darker areas on the frond located in the centre of the frond and checked for the presence of sporangia. The presence of sori in *S. latissima* is seasonal and generally restricted to autumn and winter periods; their presence hence indicates recently produced material within the old frond. Where individuals did not have visible sori present, they were divided into the other four sections specified above. All other sections were present and easily identified in all individuals, however, the relative size of the old and young fronds varied by individual. The pooled individuals differed in size; hence, they were likely of different ages or represented different developmental stages.

Specimens of *A. esculenta* were divided into the following sections: holdfast, stipe, sporophyll (thick leafy pods growing on the stipe containing sporangia), midrib and frond. All sections were present and easily identifiable in all individuals. The distinction between stipe and midrib was taken to be just below the sporophylls and care was taken to cleanly separate the frond from the midrib.

### Sample preparation

#### Total arsenic

One hundred to 200 mg of seaweed (triplicate) was accurately weighed in XP1500 vessels; 3 mL nitric acid (trace select) was added and left overnight; subsequently, hydrogen peroxide (1.5 mL) was added before microwave digestion (MARS 5, CEM) with a temperature program of 10 min ramp to 200° and held for 8 min. After digestion, the samples were diluted to 30 mL in MilliQ water [25]. The samples were stored in a freezer at − 18 °C until the day of analysis when they were further diluted by a factor of 4.

#### Sequential extraction of arsenic species

Sequential extraction was carried out to gain a mass balance of the arsenic species [19]. Sequential extraction also serves a purpose when AsLs are to be identified by organic mass spectrometry, since otherwise the signals of the AsLs may be masked under signals from other compounds. Further, it removes certain compounds which would not elute from the chosen HPLC column. Approximately 500–600 mg of seaweed (duplicate) was accurately weighed into glass vials (20 mL). Step 1: The seaweed was extracted in 2 × 5ml of hexane (left to stand for 3 and 0.5 h). The supernatants were combined and evaporated to dryness (hexane fraction). Step 2: The dry seaweed residue was subsequently extracted in 2 × 5 mL of DCM/MeOH (2:1), gently shaken until the sample was suspended in the solution and left to stand overnight (30 min for second extraction), supernatants combined and evaporated to dryness (MeOH fraction). Step 3: The residue was extracted in 15 mL of water (in plastic vials) and shaken thoroughly and left overnight to extract. Samples centrifuged at 3500 rpm for 10 min and the supernatant was removed (water fraction). Step 4: The residue was washed with 2 × 10 mL aliquots of water (as discussed in detail in Pétursdóttir et al. [19]) prior to drying in oven (60 °C). A subsample of the residue (~ 250 mg) was left overnight in 1 mL acid in the digestion vessel (XP1500) and 1.5 mL H_2_O_2_ was added prior to microwave digestion. The samples were diluted to 10 mL with MilliQ water (residue).

These four fractions represent the non-polar AsLs (hexane), the polar AsLs (MeOH fraction), the water-soluble arsenicals (water fraction) and non-extractable arsenic left in the residue. These fractions should account for the total arsenic giving further confidence in the quantification methods of the AsLs. The AsLs investigated with speciation analysis in addition to total As of the fraction were in the DCM/MeOH fraction.

#### Preparation for total As and As speciation analyses after sequential extraction

The hexane fraction from step 1 was re-dissolved in approx. 1 mL hexane and transferred to digestion bombs and evaporated before microwave digestion (1 mL of nitric acid and 1.5 mL hydrogen peroxide diluted to 10 mL after microwaving). The MeOH fraction from step 2 was re-dissolved in MeOH (approx. 1 mL) and transferred to pre-weighed HPLC vials. One subsample (100 μL) was taken for microwave digestion for total arsenic measurement (3 mL of nitric acid and 1.5 mL hydrogen peroxide, diluted to 30 mL). A second subsample was taken (300 μl) and centrifuged at 13000 rpm for 5 min on the day of speciation analysis (HPLC condition A). One millilitre of the water fraction from step 3 was digested (1 mL HNO_3_, 1.5 mL H_2_O_2_) and diluted to 10 mL.

This sequential extraction was repeated, where the main difference was an hour of mechanical shaking that was added before each extraction steps 1–3. The subsample of the MeOH fraction was transferred to an HPLC vial and evaporated to dryness under N_2_ and shipped dry under N_2_ to the University of Aberdeen for speciation analysis (HPLC condition B). The samples were shipped dry under N_2_ as it is the authors’ experience that the AsPLs are more stable when stored dry, although this has not been quantified. These sample preparations were carried out in single replicates due to limited sample material for some sections and time-consuming instrumental analysis. Additionally, triplicates were prepared for 1 sample of *A. esculenta* and 1 sample of *S. latissima* to estimate the reproducibility.

#### Extraction of arsenosugars

One hundred milligrams of each seaweed sample (triplicate) was accurately weighed into 50-mL plastic vials and shaken thoroughly with 10 mL of water and left overnight, samples were centrifuged (3000 rpm for 10 min) and the supernatant (AsSugar extract) was then transferred to a 15-mL vial (Sarstaedt). On the day of measurement, 500 μl of each AsSugar extract was transferred to plastic Eppendorf vials and centrifuged at 13000 rpm for 5 min. The supernatant was then transferred to a plastic HPLC vial.

## Analysis

### Instrumental setup and analysis

The ICP-MS was optimised daily for sensitivity and stability. ICP-MS Agilent 7500ce and 1200 Agilent HPLC were used for all measurements except for speciation of AsLs with condition B.

#### Total arsenic

Samples were analysed in no gas and He mode (1500 W). Monitored masses were *m*/*z* 75 for As, internal standards (IS) *m*/*z* 125 for Te (50 ppb), *m*/*z* 115 for In (2 ppb), and the possible chloride interference (40Ar 35Cl^+^) on *m*/*z* 75 was checked on *m*/*z* 77 (40Ar 37Cl^+^ or 77Se) and on *m*/*z* 82 (82Se). An external calibration was used for the quantification of total arsenic (beginning and end of each run) and an intermediate standard was repeated throughout the analysis. A blank was analysed between each set of triplicates.

#### Speciation AsLs

A) A C18 HPLC column (Agilent Eclipse XDB C18 5 μm, 4.6 × 150 mm) was used to achieve separation of the compounds during speciation analysis (MeOH fraction), and a flow splitter (3:1, 3 parts waste, 1 part ICP-MS) was used to reduce the volume of solvent entering the spray chamber. An internal standard of Tellurium (50 ppb) and Ge (2 ppb) was added online between flow splitter and ICP-MS to monitor instrument stability. A response factor was calculated by switching to an internal standard containing DMA (50 ppb) and Te (50 ppb) and Ge (2 ppb), injecting a blank and monitoring the measured As intensity over the course of the run as described in Amayo et al. [26]. Quantification was carried out using Ge as IS. Standards were prepared by diluting dimethyl arsenic in water and were analysed before and in the middle of the sample sequence. HPLC gradient program and parameters were specifically optimised for separation on the ICPMS 7500ce (not shown). Sample injection volume was 30 μL, 1 mL min^−1^ flow rate using a gradient program 20 min 70–100% B hold for 20 min. Eluent A is 0.1% FA in H_2_O, and eluent B is 0.1% FA in MeOH, with the ICP-MS in organic mode (1530 W), with platinum cones and 14% oxygen.
B) HPLC (Agilent 1290) coupled to Agilent 8800 (QQQ) ICPMS. Same column as in A, injection volume 0.1 mL, flow 1 mL min^−1^, gradient 0–20 min: 0–100% B, hold 25 min (eluents same as in A). Flow split post column 3:1, 3 parts to ESI-MS (LTQ Orbitrap Discovery; Thermo Scientific) and 1 part to ICP-MS. Agilent 8800 (QQQ) was used in organic mode with platinum cones and 6% optional oxygen. The As75 was analysed in oxygen mode on m/z 91, other masses monitored, P31, S32 and Ge74. The ESI-MS was operated in positive mode with a scan range of *m*/*z* 100–1300. Other parameters for the Orbitrap were 4.5 kV spray voltage, 35% normalised collision energy, 300 °C capillary temperature and 42 V capillary voltage with a resolution of 30.000 for MS and MSMS spectra. Parameters are similar to Petursdottir et al. [18].

#### Arsenolipid identification

Identification was carried out by searching for known AsL masses (Thermo, Excalibur) and matching the found *m*/*z* for the individual AsLs with the signal from the ICP-MS (*m*/*z* 75), Fig. 3. Only the major peaks had corresponding MS^2^ data. Acceptable Δ*m*/*z* (ppm) was taken at 2 ppm, but peaks under 3 ppm are also shown, Table 3.

#### AsSugars

Strong anion column (Hamilton PRPX-100 10 um 250 × 4.1 mm) with 1 mL min^−1^ of mobile phase ammonium carbonate (30 mM, pH 9) was used for the AsSugar analysis. ICPMS is in no gas mode (1500 W); masses monitored are as follows: *m*/*z* 75 for As, *m*/*z* 125 for Te (50 ppb), *m*/*z* 115 for In (2 ppb), *m*/*z* 77 and *m*/*z* 82 [18].

## Results and discussion

### Quality control

Certified reference materials (CRM) Hijiki NMIJ 7405-a (*n* = 12) and Tort-2 (Lobster Hepatopancreas) (*n* = 9) were digested and measured for totAs with each measurement showing good agreement with certified values: 22.1 ± 1.8 mg kg^−1^ for TORT-2 (cert. 21.6 ± 1.8 mg kg^−1^) and 35.9 ± 3.4 mg kg^−1^ for Hijiki (cert. 35.8 ± 0.9 mg kg^−1^). Hijiki (7405-a) was extracted alongside the samples during the sequential extractions and AsSugar extraction to monitor the extraction efficiency. The mass balance for Hijiki was in good agreement with the certified totAs in the sample, or 92–104%, Table 1. LOD and LOQ were calculated as 3× and 10× SD of blank samples, respectively, where an average dilution factor was used to convert to concentration in sample.

**Table 1:** Total arsenic of each fraction and the total arsenic of the different seaweed sections in milligrams/kilogram. Short names for *Saccharina latissima* starts with SL and for *Alaria esculenta* with AE

Short name	Section	Batch	*n*	Hexane	MeOH	Water	Residue	Sum	Total As	% recovery
SLOF	Old frond	a	2	< LOQ	7.7 ± 0.2	95 ± 9	11.8 ± 0.8	114 ± 8	117 ± 9	97
		b	3	0.49 ± 0.04	8.4 ± 0.6	97 ± 2	10.4 ± 0.7	116 ± 2		98
SLYF	Young frond	a	2	0.4 ± 0.1	8.8 ± 0.5	73 ± 3	45 ± 8	128 ± 11	116 ± 6	110
		b	1	0.45	12.0	90.9	23.6	126.9		110
SLSP	Sori	a	2	0.7	11 ± 1	126 ± 6	21 ± 11	159 ± 5	114 ± 28	139
		b	1	1.1	12.5	109.6	5.6	128.8		113
SLHF	Holdfast	a	2	< LOQ	5.2 ± 0.4	94 ± 9	11 ± 1	110 ± 8	127 ± 6	87
		b	1	0.11	6.0	112.4	9.3	127.8		101
SLS	Stipe	a	2	< LOQ	2.9 ± 0.3	40.1 ± 0.1	8 ± 1	51 ± 1	53 ± 5	97
		b	1	0.03	4.8	36.7	6.8	48.3		91
AES	Stipe	a	2	0.38 ± 0.03	3.7 ± 0.5	33.3 ± 0.1	4.1 ± 0.3	41.4 ± 0.4	53 ± 3	78
		b	1	0.15	6.5	31.4	4.5	42.5		80
AEMR	Midrib	a	2	0.48 ± 0.03	4.1 ± 0.2	30 ± 2	9.6 ± 0.6	46 ± 2	43 ± 4	104
		b	3	0.33 ± 0.17	4.1 ± 0.2	29 ± 1	6.0 ± 0.5	39.4		92
AESP	Sporophyll	a	2	0.4 ± 0.4	8.0 ± 0.6	102 ± 3	12 ± 2	128 ± 4	116 ± 6	106
		b	1	0.11	13.2	92.7	6.4	112.4		97
AEHF	Holdfast	a	2	0.03 ± 0.02	7.3 ± 0.6	82 ± 4	6 ± 2	96 ± 3	102 ± 2	94
		b	1	0.78	8.7	80.1	5.1	94.7		93
AEF	Frond	a	2	0.70	4.0 ± 0.1	85 ± 5	16 ± 1	106 ± 6	93 ± 4	115
		b	1	0.56	4.6	64.4	10.9	80.4		87
Hijiki	Hijiki	a	2	LOQ	5.6 ± 0.3	20 ± 1	7.1 ± 0.1	33 ± 1	35.6	92
		b	2	0.10 ± 0.01	10.1 ± 0.7	20 ± 2	6.8 ± 1.0	37 ± 2		104

LOD 0.005 mg kg^−1^, LOQ 0.02 mg kg^−1^

### Total arsenic and mass balance

The total arsenic of each step of the sequential extraction was measured for totAs to account for the mass balance of the method. The sum of all four steps (hexane, MeOH/DCM, water, residue) showed good agreement with the totAs in the seaweed where the recovery ranged generally from 78 to 115%; an exception was the sori where the recovery was higher (139%), Table 1, most likely due to overestimation of the water and residue fractions. The amount of arsenic in the hexane fraction seemed more arbitrary than in the other fractions, ranging from very little non-polar As present to up to 1 mg kg^−1^. CRM Hijiki has been reported before with reasonable agreement with the totAs for the MeOH/DCM phase at 6.24 mg kg^−1^ [20] and 3.8 mg kg^−1^ [27] compared to 7.9 mg kg^−1^ here on average, Table 1. The lower value reported by Wolle et al. [27] for the MeOH/DCM phase is likely due to a water extraction prior to the MeOH/DCM as reported in Pétursdóttir [19] and also speculated by Wolle et al. [27]. The totAs for the hexane fraction [20] was higher than found here, or 0.97 mg kg^−1^ compared to 0.1 mg kg^−1^. Total As of water-soluble fraction of hijiki was in good agreement at 22.6 mg kg^−1^ [27] compared to 20 ± 2 mg kg^−1^ found here, Table 1.

The total arsenic was measured for each section of the two seaweed species; there was a trend for concentrations in stipe and/or midrib to be only half of those observed in the other seaweed parts, Table 1, indicating that less arsenic is stored within the stipe than other parts of the seaweed.

For S. latissima, the highest amount of non-polar As was present in the sori and the young frond, but for *A. esculenta*, in the frond and midrib. The samples were extracted in two batches prepared and analysed on separate days; in general, batch A extracted less of polar As than batch B, where mechanical shaking for batch B may have contributed to the better extraction. There was more As in the residue for batch A. Most likely, the sample residue was not rinsed well enough before drying; hence, any remaining As from the previous step (water extraction) could lead to an overestimation. In general, there was good consensus on the batches showing good repeatability, Table 1.

### Arsenolipids

**Fig. 2:**
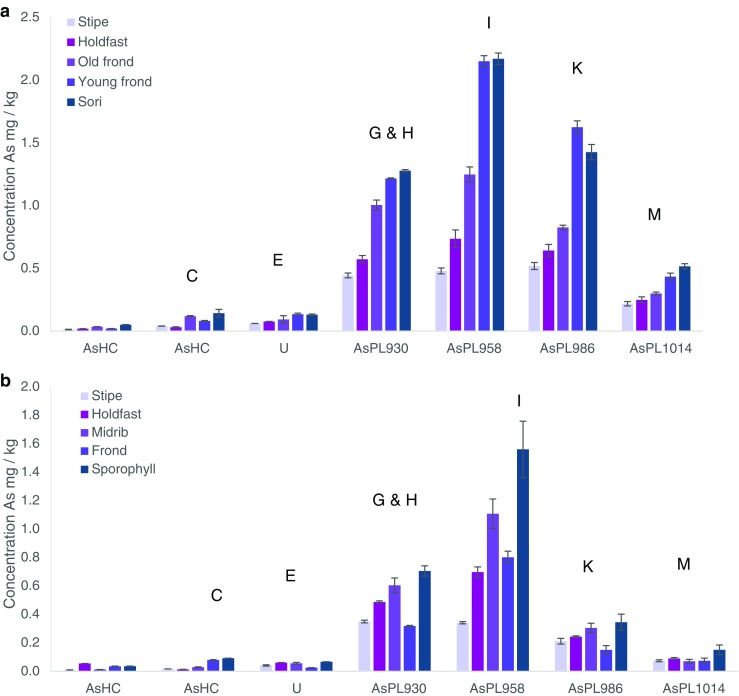
Quantification of AsLs (batch A) for **a***Saccharina latissima* and **b***Alaria esculenta*. Quantification is given in ESI. Letters correspond to equivalent peaks in batch B (e.g. Fig. 3). The major AsL per each peak is given

When the samples were collected and sectioned, the working hypothesis was that seaweed parts differed not only in age, but also in biological function or developmental stage and therefore differences in the AsL composition could be expected. Previously, it had been shown, for example, that alginate composition varied by tissue type reflecting the different biological functions [28]. These findings influenced how the algae were sectioned in this study. Both holdfast and stipe (functions: anchorage/attachment and support, respectively) of *S. latissima* represented older and more rigid structures, while the blade is renewed on an annual basis. However, holdfasts contained a visible mixture of both old and newly produced rhizoids entwined together. The stipe also contained younger growth since the inner part of the stipe is older and the outer part is younger. The internal profile of the stipe and holdfast was not investigated since for this, e.g. bioimaging would be needed; However, as kelp stipe and holdfasts have distinct functions, their chemistry, in particular their alginate composition, is likely to differ, and is additionally influenced by season and environment [[28], [29, 30]]. The frond was clearly divided into older and newer sections, with an age gradient along the blade and youngest parts towards the stipe, and oldest towards the blade tip. The sori, produced seasonally, represented reproductive materials and were visibly different as a dark patch containing sporangia. Therefore, a distinction was made both for age and metabolic function of the frond. The first results from the MeOH fraction (batch A) showed a significant difference between the different seaweed segments. By ordering the segments by age, function or reproductive stage, an increasing trend can be observed, which is especially pronounced for *S. latissima* with younger or reproducibly active tissue containing some of the AsSugPLs in significant higher amounts than older thallus parts. *A. esculenta* also contains more AsLs in the reproductive tissue than elsewhere, Fig. 2.

The ESI-MS analysis was performed a week later than the quantification and due differences in the retention times only the main AsPLs could be assigned based on data from CRM hijiki (see Electronic Supplementary Material (ESM) Section 2). The AsPL do not appear to be stable with time; hence only the most abundant were still present in the samples. The sample preparation was repeated (batch B) and measured the second time with simultaneous HPLC-ICP-MS/ESI-MS for reliable and accurate identification of AsLs. This allowed for the verification of the quantification and the noticed trend (ESM Section 5). Even though the concentration of the AsLs was lower for the first batch of samples, the increasing trend of AsPL with ‘activity’ was seen for the main AsPLs in *S. latissima*, although the trend was not noticeable for the heavier AsPLs. This may be due to their lower concentration, or potentially they are less stable, ESM Fig. S11. The quantification for the *A. esculenta* indicates a similar trend for the AsPLs but the results were more ambiguous due to instrumental problems (ESM Section 5).

**Fig. 3:**
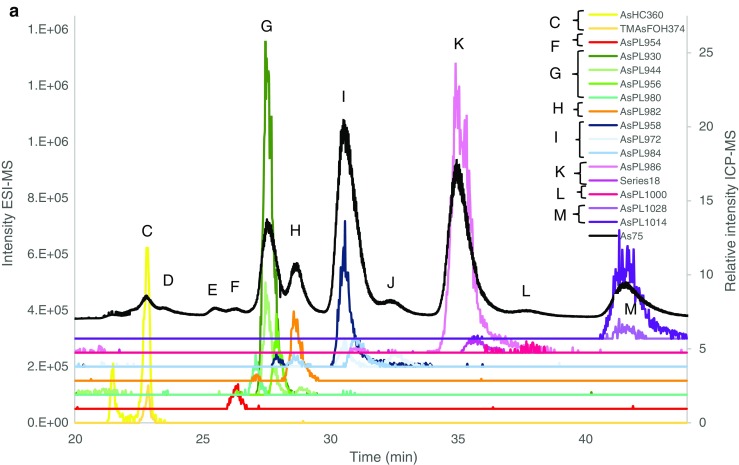

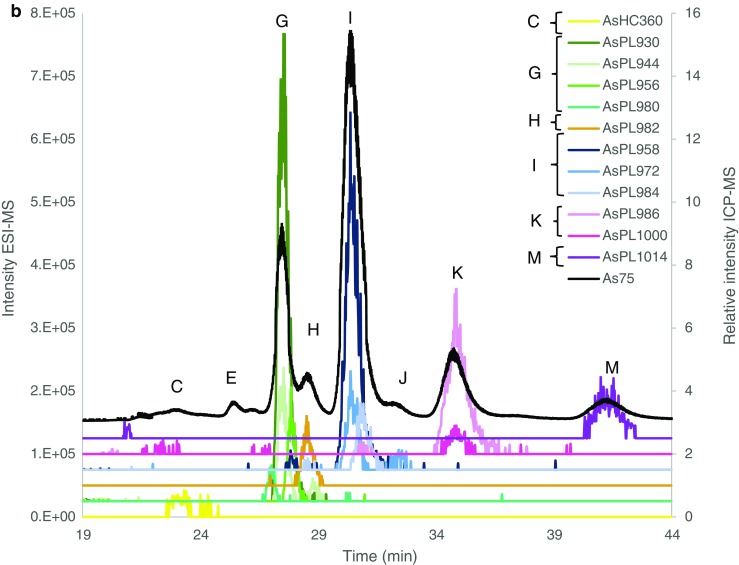

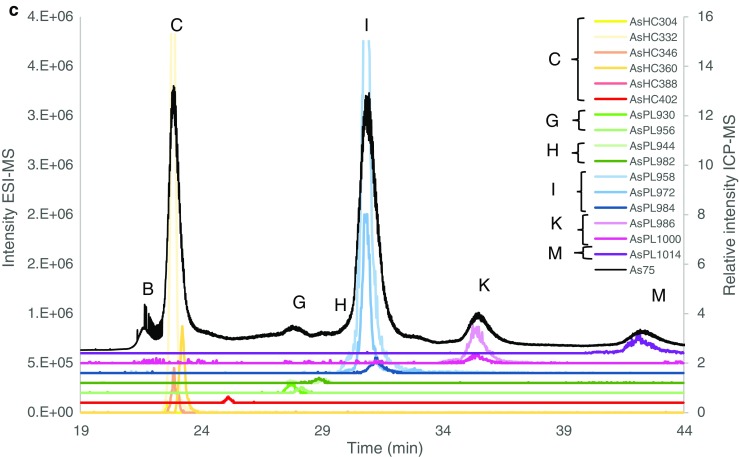
Overlay of ESI-MS data (coloured lines) and ICP-MS (black, *m*/*z* 75) for **a***Saccharina latissima* young frond, **b***Alaria esculenta* midrib and **c** Hijiki CRM 7405a. There was a minor time delay for the ESI-MS data and to compensate this, the ICP-MS signal was shifted to a 0.7–0.9-min earlier retention time for all chromatographs. Magnification of close-eluting peaks distinguished with ESI-MS is shown in ESM Fig. S5

Quantification for both batches was in good agreement with the totAs of the extracts, where the column recovery was 96 ± 11% and 85 ± 11% for batches A and B, respectively. CRM NMIJ 7405-a (Hijiki) was analysed with all AsL measurements. Hijiki is not certified for AsLs but Glabonjat et al. [20] report identification of 7 AsLs. The two AsHCs identified (AsHC332—1.07 ± 0.04 mg kg^−1^, AsHC360—0.090 ± 0.005 mg kg^−1^) in their work are in good agreement with the main AsHC identified here (AsHC332 major, AsHC360 minor, TMAsFOH minor, other AsHCs traces), Fig. 3c. Batch A showed good agreement with the quantification (1.11 ± 0.05 mg kg^−1^) for the AsHCs but batch B had higher concentrations (2.0 ± 0.1 mg kg^−1^), Table 2. The peak pattern for AsPLs is similar, Fig. 3c, as reported by Glabonjat et al. [20] (AsPL958 (here peak I) 1.59 ± 0.03, AsPL986 (here peak K) 0.304 ± 0.006, AsPL1014 (here peak M) 0.21 ± 0.01, AsPL1042 0.114 ± 0.003, AsPL1070 0.043 ± 0.002), although the two heavier AsPLs were not found here due to the chromatograph being cut short before their elution. Quantification for peaks K and M was in good agreement with the reported values for AsPL986 and 1014, Table 2; however, peak I contained higher concentration than reported for the AsPL958. Additionally, to these main 3 AsPLs, a more diverse flora of AsPLs were either identified here as minor components or found as traces, Fig. 3c.

**Table 2:** Quantification of AsLs in *Saccharina latissima* and *Alaria esculenta* (mg kg^−1^), batch B (data for batch A in ESM) *n* = 1 for all but hijiki (*n* = 2), *Saccharina latissima* old frond and *Alaria esculenta* midrib (*n* = 3)

	Saccharina latissima						*Alaria esculenta*					Hijiki	
Peak	Rt (min)	Stipe	Holdfast	Old frond	Young frond	Sori	Rt (min)	Stipe	Holdfast	Midrib	Sporophyll	Rt (min)	
A	2.6	0.51	1.3	3.0 ± 0.3	3.3	4.1	2.5	3.0	4.9	0.80 ± 0.03	6.1	2.7	0.30 ± 0.02
B	10.7			LOQ	LOQ		10.2	0.072	0.070	LOQ	0.052		
							22.6	0.046	0.070		0.087	22.6	0.30 ± 0.06
C	23.8	0.12	0.17	0.34 ± 0.02	0.17	0.17	24.0	0.068	0.093	0.06 ± 0.03	0.18	23.8	2.0 ± 0.1
D	25.5	0.072		0.061 ± 0.006	0.066	0.077							
E	26.4	0.063	0.043	0.053	0.056	0.045	26.5	0.089	0.10	0.06 ± 0.01	0.11		
F	27.2	0.051	0.049	0.047 ± 0.008	0.060	0.046	27.1			0.018 ± 0.008			
G	28.6	0.47	0.36	0.70 ± 0.09	1.0	0.94	28.5	0.56	0.90	0.58 ± 0.05	0.80	28.7	0.17 ± 0.04
H	29.7	0.20	0.26	0.41 ± 0.03	0.52	0.60	29.6			0.18 ± 0.01	0.31		
I	31.7	0.84	0.96	1.2 ± 0.1	2.5	2.2	31.7	0.70	1.2	1.3 ± 0.2	2.5	31.8	2.3 ± 0.2
J	33.4	0.09	0.11	0.11 ± 0.01	0.17	0.17	33.2			0.06 ± 0.01			
K	36.2	1.3	1.1	0.95 ± 0.08	1.9	1.3	35.8	0.40	0.53	0.35 ± 0.01	0.63	36.3	0.37 ± 0.09
L	38.8	0.12	0.11	0.10 ± 0.01	0.064	0.10							
M	43.0	0.47	0.34	0.30 ± 0.02	0.65	0.40	42.2	0.17	0.18	0.16 ± 0.01	0.31	43.1	0.20 ± 0.03
Sum		4.3	4.8	7.3	10.6	10.2		4.8	8.7	3.6	12.4		5.7

LOD 0.009 mg kg^−1^, LOQ 0.03 mg kg^−1^

Results of *S. latissima* (from Scotland) reported in Raab et al. [7] were in good agreement with regard to the AsLs identified with the addition of AsPL954, AsPL1012 and AsPL1028 found here. The AsHCs and AsFAs were the same as those identified by Raab et al. [7] except for AsHC388 which was only found here in *A. esculenta* not *S. latissima*. The AsFAs are found in such trace amounts that there is not a peak found at *m*/*z* 75 with the ICP-MS (ESM Fig. S3b). The similarity of AsLs found indicates that the AsL composition of As species in *S. latissima* varies little with location but the concentrations can vary more as the concentration of AsHCs is very low here unlike in Raab et al. [7]. Mono-acyl AsPLs are found at trace levels (mainly AsPL692 and AsPL720), ESM Table S1. These mono-acyl AsPLs (AsSugPLs) were identified in Kombu as a potential artefact resulting from the process of preparing the food product (sun-dried for days); however, they were also identified in fresh Kombu albeit at much lower concentrations [10]. Their presence here at trace levels (no MSMS data) indicates that they may be breakdown products from di-acyl AsPLs. This is supported by the fact that the mono-AsPLs identified here correspond with di-acyl AsPLs identified in the same samples, i.e. the two main mono-AsPLs (692 and 720) could be breakdown products of the two main AsPLs in *A. esculenta* and *S. latissima* the AsPL930 and AsPL958, ESM Table S1. Fittingly, the AsPL692 was not present in hijiki, which did not contain AsPL930.

Only the most abundant AsL per peak had MSMS spectra (AsHC332, AsPL930, AsPL958, AsPL986, AsPL1014) where the most commonly found fragment ion for the AsPLs was 409.024 [7] Table 3. There was no MSMS data for *m*/*z* 375.26; therefore, it is difficult to distinguish between the isomers TMAsFOH374 and AsHC374. The AsHC332, AsHC346, AsHC360 and AsHC374 are all saturated AsHCs that differ by 1 carbon in chain length where AsHC346 and AsHC360 elute with slightly different retention times but AsHC360 and *m*/*z* 374 elute at the exact same time. This is in accordance with previous identification of TMAsFOH374 [12, 19]. This could indicate that the *m*/*z* 374 is TMAsFOH374; however, AsHC332 and AsHC346 also elute at the exact time, and MSMS data reveals that *m*/*z* 332 is indeed AsHC332. The data are therefore not conclusive to confidently identify this minor species.

**Table 3:** Identified As species in *Alaria esculenta*, *Saccharina latissima* and CRM hijiki. Additional species were only found as minor traces in ESM Table S2

			Alaria esculenta					Saccharina latissima					CRM	
			Sporophyll	Stipe	Holdfast	Frond	Midrib	Young frond	Sori	Holdfast	Stipe	Old frond	Hijiki	
Peak	Short name	MH+	Δ*m*/*z* (ppm)											Calc. MH+
A	AsHC304	C15 H34 O As											− 0.63	305.1826
C	AsHC332	C17 H38 O As						1.10	1.85			− 0.43	1.40^d^	333.2139
	AsHC346	C18 H40 O As							1.60				− 1.48	347.2295
	AsHC358	C19 H40 O As											− 0.87	359.2295
	AsHC360	C19 H42 O As	1.32		− 0.81			− 1.89	− 1.15	− 1.51	− 0.98	− 1.15	0.29	361.2452
		C20 H44 O As						− 1.74	− 0.84	− 1.66	0.23	− 0.44	− 0.20	375.2608
D	AsHC402	C22 H48 O As	− 0.01			− 0.90	− 0.08	0.76	− 1.82			− 0.83		403.2921
	AsHC388	C22 H34 O As			− 0.55		− 1.65							389.1826
F	AsPL954	C45 H85 O14 As P	0.61	0.16	0.61		− 0.67	0.03	− 1.63	0.54	0.86	0.42		955.4893
G	AsPL980	C47 H87 O14 As P	0.62	− 0.81	0.50		− 0.81	− 0.93	0.62	− 0.68	− 0.06	− 0.49	− 1.99	981.5049
	AsPL930	C43 H85 O14 As P	2.98^a^	2.59^a^	− 0.03	1.67	− 1.93^a^	− 2.13^a^	1.87^a,b^	− 1.22^a^	− 1.09^a^	− 1.15^a^		931.4893
	AsPL944	C44 H87 O14 As P	1.04	0.58	1.10		− 1.23	− 1.15	1.68	− 0.77	− 0.07	− 0.51	− 1.09	945.5049
	AsPL956	C45 H87 O14 As P	1.60	1.98	1.21	0.83	1.15	2.17		0.72^4^	1.60	0.64	0.57	957.5049
H	AsPL982	C47 H89 O14 As P	1.60	0.10	0.60		1.72	2.58	− 0.40	1.78	2.81	2.46	0.54	983.5206
I	AsPL958	C45 H89 O14 As P	0.431	− 0.98	− 0.53	1.00	− 0.72^a^	− 0.53^a^	3.09^a^	0.99	− 0.27	0.10^a^	− 1.36^a^	959.5206
	AsPL972	C46 H91 O14 As P	− 0.92	− 1.55	− 0.93		− 1.55	− 1.09	− 1.80	− 0.87	0.27	− 0.42	− 1.68	973.5362
	AsPL984	C47 H91 O14 As P	− 1.91	− 0.92	0.08	− 3.09	0.38	0.88	0.20	0.16	− 0.98	0.51	1.16	985.5362
K	AsPL986	C47 H93 O14 As P		2.65	− 0.01	0.30	− 1.00^a^	1.48^a^	− 2.18^a^	− 0.90^a^	− 0.45^a^	0.23^a^	− 1.07^a^	987.5519
	AsPL1012	C49 H95 O14 As P	− 0.46	− 0.82	− 1.30			− 1.97	− 1.72	− 1.25	− 0.34	0.03		1013.568
K/L	AsPL1000	C48 H95 O14 As P		0.94	0.27			− 0.16	− 1.80	− 1.07^c^	− 0.03	0.09^c^	− 0.83	1001.568
M	AsPL1014	C49 H97 O14 As P			0.36	− 1.44	0.72	− 0.24	− 1.09	0.12	− 0.24	0.00^a^	− 0.90	1015.583
	AsPL1028	C50 H99 O14 As P	− 0.61	− 0.86				− 0.61	− 1.33	0.21	0.10	1.05		1029.599

MSMS fragments (full detail in ESI): ^a^409 ^b^391, ^c^423, ^d^102.95, 104.97, 122.98

### Arsenosugars

**Fig. 4:**
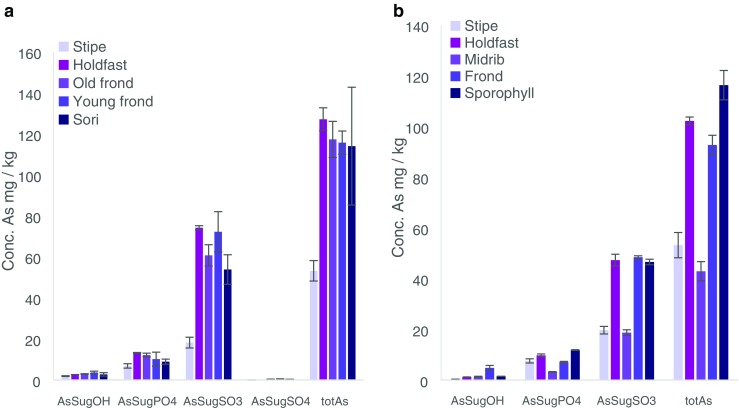
AsSugars, other As trace peaks not shown, full quantification in ESM Tables S4 and S5. **a***Saccharina latissima*. **b***Alaria esculenta*

Samples were extracted in triplicate for AsSugar quantification and identification. The quantification showed a reasonably good column recovery 87 ± 12% (range 62–103%). The main peaks in the samples were AsSugOH, AsSugPO_4_ and AsSugSO_3_ in all samples, where other peaks were only minor (DMA, unknown and AsSugSO_4_/AsV), ESM Tables S4-S6. Identification was based on an in-house reference material analysed in the same run as the samples. The identification of 4 main AsSugars (AsSugOH, AsSugPO_4_, AsSugSO_3_, AsSugSO_4_) had been previously done for this sample with LC-MS/MS (Thermo, Quantiva). Spiking of the samples also revealed the retention time of iAs and DMA. As(III) elutes with AsSugarOH and As(V) with AsSugarSO_4_. Low quantities of iAs were expected as, e.g. reported in Pétursdottir et al. [31] where the iAs was below 0.3 mg kg^−1^ in 10 samples of Icelandic *S. latissima*, and for the *A. esculenta*, the iAs cannot be significant since no peak was found at the Rt of As(V) and AsSugSO_4_ and small peaks at Rt for As(III) and AsSugarOH (Fig. 4b). Quantification of AsSugars in CRM Hijiki 7405a has been reported by Wolle et al. [27], showing good agreement with the quantification here, ESM Table S6, although double concentration of AsSugarSO_3_ was found here compared to Wolle et al., where this higher concentration may possibly be attributed to co-elution of other water-soluble species.

For the AsSugars, there was no noticeable trend in concentration based on the different sections of the seaweed except for the similarity of AsSugSO_3_ to the totAs, i.e. low conc. for stipe and midrib, and a much higher and similar concentration for all other parts of the seaweed, Fig. 4.

## Conclusions

In depth, arsenic speciation using parallel HPLC-ICP-MS/ESIMS showed that both *A. esculenta* and *S. latissima* were very rich in AsLs, where the vast majority was in the form of diverse AsPLs. Very little was found as AsHCs, differing from hijiki which had approximately equal amounts of AsHCs and AsPLs. There are no toxicological data on AsPLs yet and these are urgently needed to better estimate the risk derived from, e.g. human consumption of seaweed.

Employing sophisticated speciation analyses further unravelled new information about AsLs in seaweed. Arsenic was not found to be uniformly distributed within the two brown macroalgal species, with lower levels of totAs found in the stipe/midrib compared to other thallus parts. This trend is mainly due to AsSugSO_3_ which was the most abundant As species in the two species. The AsLs however had a different distribution to the AsSugars or the totAs where the AsPLs differed by approx. a factor of 4 between the sections containing the lowest and highest concentration of AsPLs. When the sections were placed in order of estimated metabolic activity also incorporating an estimate of tissue age, taking into consideration within-blade variation and the fact that stipe and holdfast contained the oldest seaweed parts due to their perennial nature, there appeared to be a relationship between the metabolic activity and AsPLs, with lower levels of AsPLs in oldest parts. All seaweed materials had been collected at the time of the highest growth rates (late winter), and when newly formed patches of sori on *S. latissima*, and sporophylls on *A. esculenta*, were present. The data therefore suggest either that the two kelp species preferentially store the AsPLs in younger/metabolically active thallus parts, or that they were producing these AsPLs, possibly as a side product of a natural biological activity. Subsequently, since there is no evidence for a physiological requirement (or function) of these potentially toxic species, it seems they break down to AsSugars. This would suggest that AsPLs are formed before AsSugars or, alternatively, they are formed from AsSugars which undergo a temporary cycle to produce AsPLs. Currently, neither the sequence of biochemical processes involved, nor their potential biological relevance and energy requirements are clear.

Lai et al. [32] showed that different extraction efficiency of total As for *Fucus spiralis* depended on the season and they speculate that the observed lower extraction efficiency in winter (February) could be due to either different distribution of arsenic species (i.e. not as water-soluble arsenic as in summer) or that the structure of the plant changes hindering extraction of water-soluble arsenic. It should be noted that the seasonal growth pattern of Fucales differs from that of Laminariales (including *S. latissima* and *A. esculenta*) in that fucalean metabolic activity is lowest during winter [33, 34]. This current work indicates the possibility of a different distribution of As in winter; i.e. when the kelp species were actively growing [35], the As is predominantly accumulated as AsLs rather than water-soluble arsenic. This would thus be in agreement with Lai et al. [32] who further suggested that this distribution was related to thallus age, with higher proportions of water-soluble arsenic in older than in younger samples.

## Electronic supplementary material

ESM 1 (PDF 3408 kb)
