# Supplementary material for: Arsenolipids are not uniformly distributed within two brown macroalgal species Saccharina latissima and Alaria esculenta
Source: Anal Bioanal Chem. 2019 May 31;411(19):4973–85. doi: 10.1007/s00216-019-01907-x (PMC6611760; doi:10.1007/s00216-019-01907-x)
Supplement: Supplementary file 1 — (PDF 3408 kb) [file 216_2019_1907_MOESM1_ESM.pdf]

Analytical and Bioanalytical Chemistry

Electronic Supplementary Material

Arsenolipids are not uniformly distributed within two brown macroalgal species *Saccharina latissima* and *Alaria esculenta*

Ásta. H. Pétursdóttir, Jonathan Blagden, Karl Gunnarsson, Andrea Raab, Dagmar Stengel, Jörg Feldmann, Helga Gunnlaugsdóttir

Contents

Quantification batch A 3

Identification batch A. 4

Identification batch b 7

  Minor traces 7

  MSMS data from Excalibur 11

Instability of AsPLs? 17

Comparison of the two extractions of the (same) samples 18

  Alaria esculenta 18

  Saccharina latissima 20

Arsenosugars 22

Information:

Batch A: Samples prepared and measured at Matís, Iceland (ICPMS). Shipped to Abdn for identification (ESI-MS). Identification not fully conclusive.

Batch B: Samples prepared again (there was still enough material for another analysis). Samples prepared at Matís, shipped dry (under N2). Re-dissolved and measured at Abdn with parallel ICP-MS/ESI-MS.

Raw data: The identification data (.raw) is found in open data repository Zenodo at https://urldefense.proofpoint.com/v2/url?u=https-3A__zenodo.org_communities_sos_&d=DwIGaQ&c=vh6FgFnduejNhPPD0fl_yRaSfZy8CWbWnIf4XJhSqx8&r=lI2nPFBwmKsJcsgWdEIxRA&m=dYSZ1Wajh7IilAyUK0bJHw-FdAzS8XhXMQKaf0voNMY&s=AGqvNJN3hmC6M93Hoor42aNTC8YxY3t7SLGdknhZSjQ&e=

DOI for the identification data is 10.5281/zenodo.2671494.

Quantification batch A

Table S1 Quantification of AsLs in Saccharina latissima and Alaria esculenta (mg kg^{-1}), batch a), (n=2)

		**Saccharina latissima**						**Alaria esculenta**					**Hijiki**	
**Peak**	**Rt (min)**	**Stipe**	**Holdfast**	**Old frond**	**Young frond**	**Sori**	**Rt (min)**	**Stipe**	**Holdfast**	**Midrib**	**Frond**	**Sporophyll**	**Rt (min)**	
A	*3.1*	0.68 ± 0.07	2.25 ± 0.01	4.1 ± 0.6	4.1 ± 0.5	5.9 ± 0.1	*3.1*	2.4 ± 0.1	5.2 ± 0.1	1.09 ± 0.08	1.67 ± 0.02	5.3 ± 0.5	*3.2*	0.44 ± 0.04
B	*16.6*	0.012 ± 0.002	0.019 ± 0.001	0.035 ± 0.001	0.020 ± 0.001	0.051 ± 0.002	*16.5*	0.010 ± 0.001	0.054 ± 0.001	0.012 ± 0.001	0.035 ± 0.002	0.036 ± 0.001	*17.0*	0.26 ±0.02
C	*20.2*	0.040 ± 0.001	0.030 ± 0.004	0.119 ± 0.004	0.08 ± 0.01	0.14 ± 0.03	*21.3*	0.02	0.013 ± 0.001	0.029 ± 0.002	0.079 ± 0.003	0.092 ± 0.001	*20.9*	1.11 ± 0.05
D	*24.7*	0.061 ± 0.02	0.076 ± 0.001	0.09 ± 0.03	0.134 ± 0.004	0.13 ± 0.01	*24.7*	0.041 ± 0.001	0.061 ± 0.001	0.056 ± 0.006	0.025 ± 0.001	0.066 ± 0.001	*23.4*	0.15
E	*26.3*	0.44 ± 0.02	0.57 ± 0.03	1.00 ± 0.04	1.22 ± 0.01	1.28 ± 0.01	*26.4*	0.35 ± 0.01	0.49 ± 0.01	0.60 ± 0.05	0.318 ± 0.004	0.70 ± 0.04	*26.2*	0.26 ± 0.03
F	*28.3*	0.48 ± 0.02	0.74 ± 0.07	1.25 ± 0.06	2.15 ± 0.04	2.17 ± 0.05	*28.5*	0.34 ± 0.01	0.70 ± 0.04	1.1 ± 0.1	0.80 ± 0.04	1.6 ± 0.2	*28.0*	3.2 ± 0.04
G	*31.2*	0.52 ± 0.02	0.64 ± 0.05	0.83 ± 0.05	1.62 ± 0.05	1.43 ± 0.06	*31.5*	0.21 ± 0.02	0.24 ± 0.01	0.30 ± 0.03	0.15 ± 0.03	0.34 ± 0.06	*30.7*	0.40 ± 0.07
H	*35.1*	0.22 ± 0.02	0.25 ± 0.02	0.30 ± 0.01	0.43 ± 0.03	0.52 ± 0.02	*35.4*	0.07 ± 0.01	0.090 ± 0.007	0.07 ± 0.01	0.074 ± 0.02	0.15 ± 0.03	*34.6*	0.24 ± 0.06
I													*40.2*	0.12 ± 0.02
Sum		*2.5*	*4.6*	*7.7*	*9.8*	*11.6*		*3.5*	*6.8*	*3.3*	*3.2*	*8.3*		*6.1*

Identification batch A

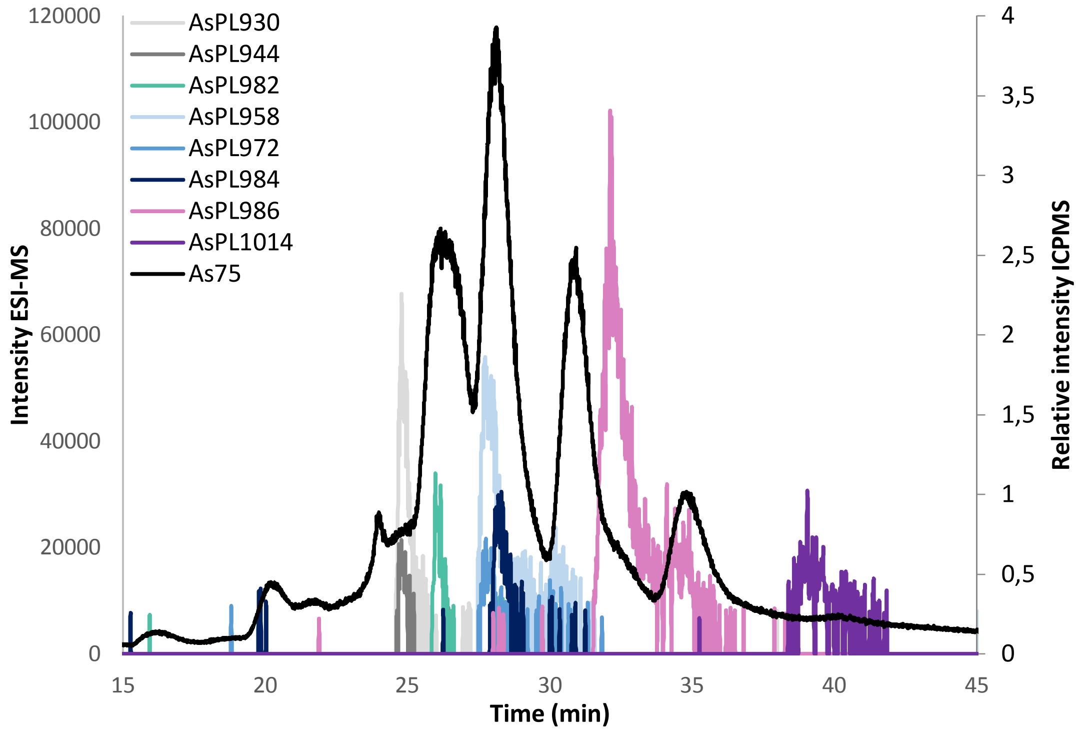

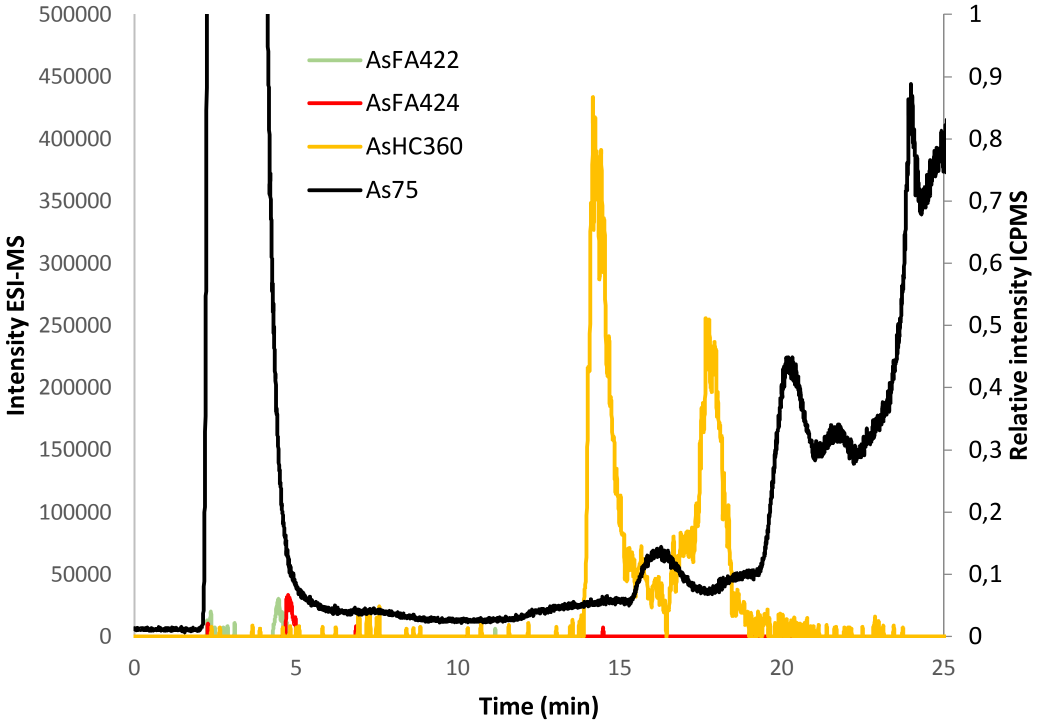

Fig. S1 Example chromatogram for batch A of elution of identified peaks. S. latissima old frond. As75 analysed at Matís (ICPMS) and other m/z analysed at University of Aberdeen a week later (ESI-MS). Same type of column, but not the exact same column

The elution order of species is the same with ESI-MS and ICPMS, but the retention times have shifted due to using columns of different batches and slightly different length of connections between instruments. Peak allocation was made by comparison to Hijiki CRM that was analysed in the same

run and which is reported in Glabonjat et al [1] as well as comparison to batch B. When batch A was analysed the intensity for AsHC360 was much higher than for the AsPLs, whereas this was not the case for batch B. This may have to do with the AsPLs possibly being less stable than the AsHCs (the identification was carried out a week later than quantification). The AsFAs elute in the void volume since the starting gradient was at 70% MeOH. The separation is worse than at Abdn (figure 3 in manuscript), even though this gradient program showed the best separation after optimisation of gradient programs. Problems with separation may be attributed to the specific column, but since other C18 and C8 columns also showed poor separation (where in one case the exact same column had worked in other laboratories) it was possible the fault was e.g. with the HPLC, i.e. the mixing of the gradient solvents was somehow different leading to poorer results. The HPLC was mainly used previously isocratically.

Split peak pattern for AsHC360 has e.g. been reported by Pétursdóttir et al [2].

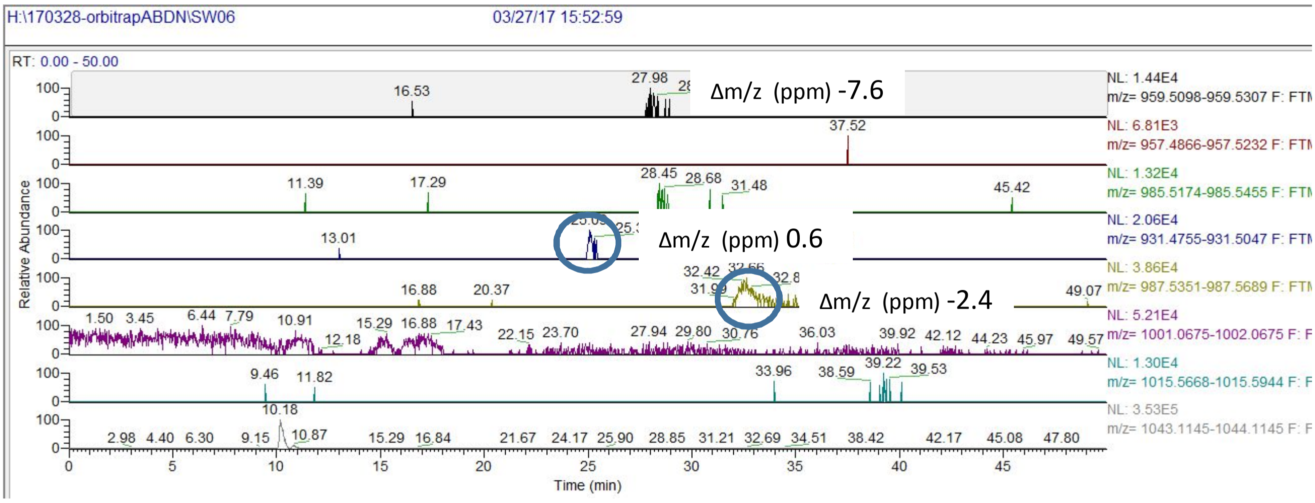

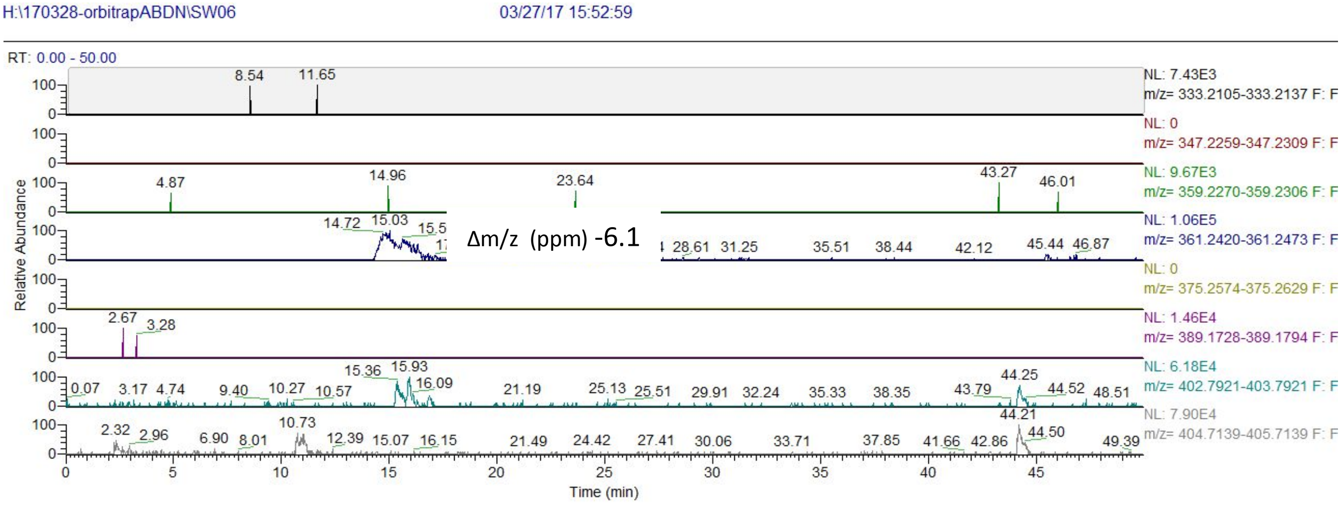

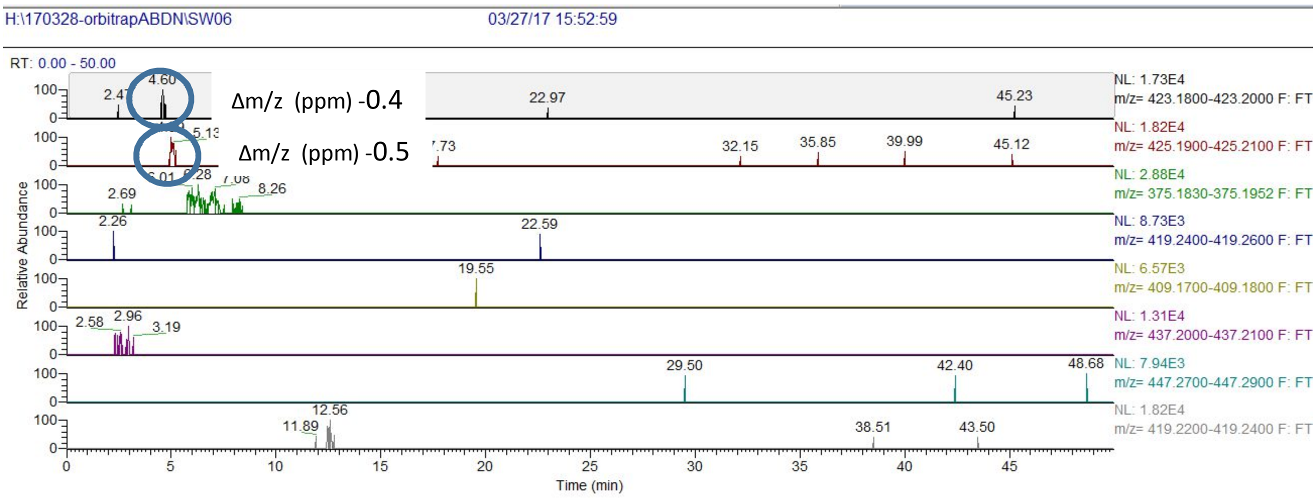

Fig. S2 Identified peaks in S. latissima stipe as seen in software. Batch a

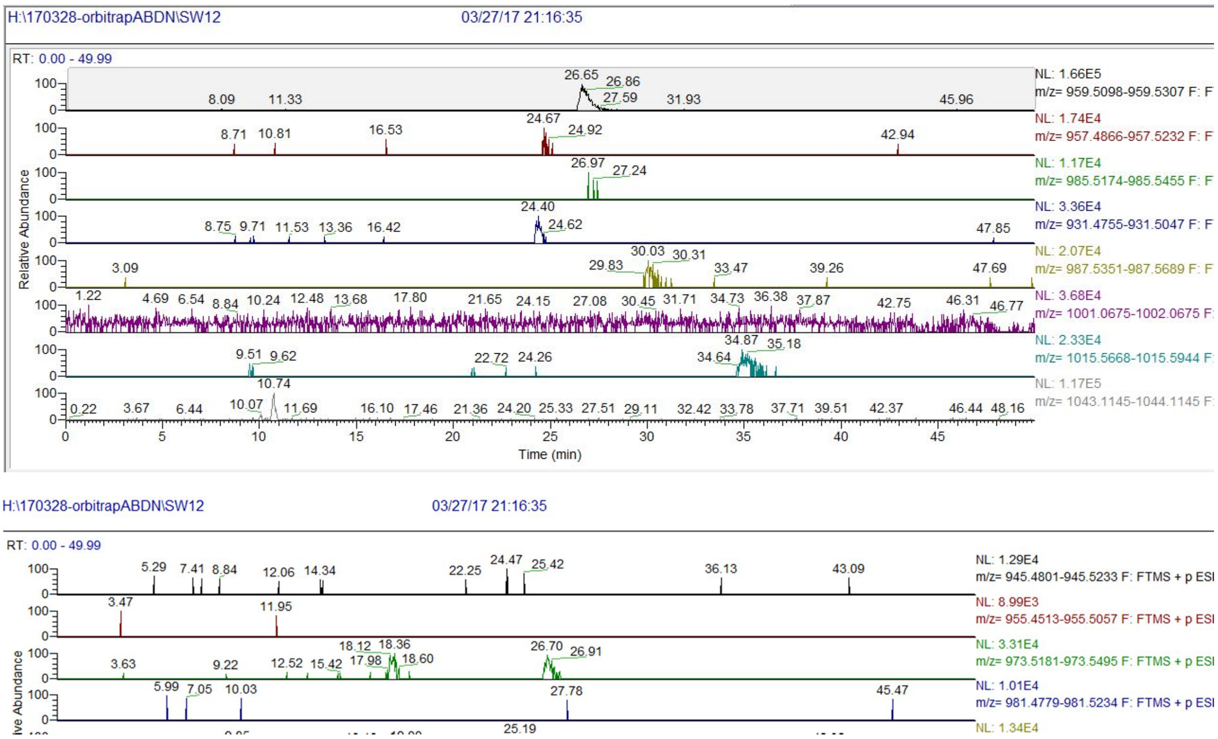

Fig. S3 Identified peaks in Hijiki as seen in softare. Batch a

Identification batch B

Minor traces

Table S2 Identification of AsL groups only as minor traces (batch b)

Rt (min)	Short name	Mol formula MH+	Alaria esculenta: Sporo-phyll	Stipe	Hold-fast	Frond	Midrib	Saccharina latissima: Young frond	Sori	Hold-fast	Stipe	Old frond	CRM: Hijiki	Break down from?
	AsFA422	C22 H36 O3 As					0.16	-1.71	1.58	-2.46	-0.03			
18-19	AsFA424	C22 H38 O3 As						-1.49	4.70		-0.90	3.12		
	AsFA374	C18 H36 O3 As					0.77							
	AsPL692	C27 H55 O13 As P	-1.47		-1.83		-0.33	-1.29	-1.65	-0.33	-2.09	-0.59		AsPL930
	AsPL742	C31 H57 O13 As P							-2.15					AsPL958
20-22	AsPL720	C29 H59 O13 As P	-0.22	-1.83	-1.06	-1.15	-1.23	-0.90	-1.48	-0.47	-0.81	-1.48	-0.90	AsPL972
	AsPL734	C30 H61 O13 As P							1.338		2.25			AsPL980
	AsPL746	C31 H61 O13 As P							-0.24	1.32		-2.86		AsPL982
	AsPL748	C31 H63 O13 As P									-1.49		-1.33	AsPL984
23	AsPL776	C33 H67 O13 As P								-0.79				

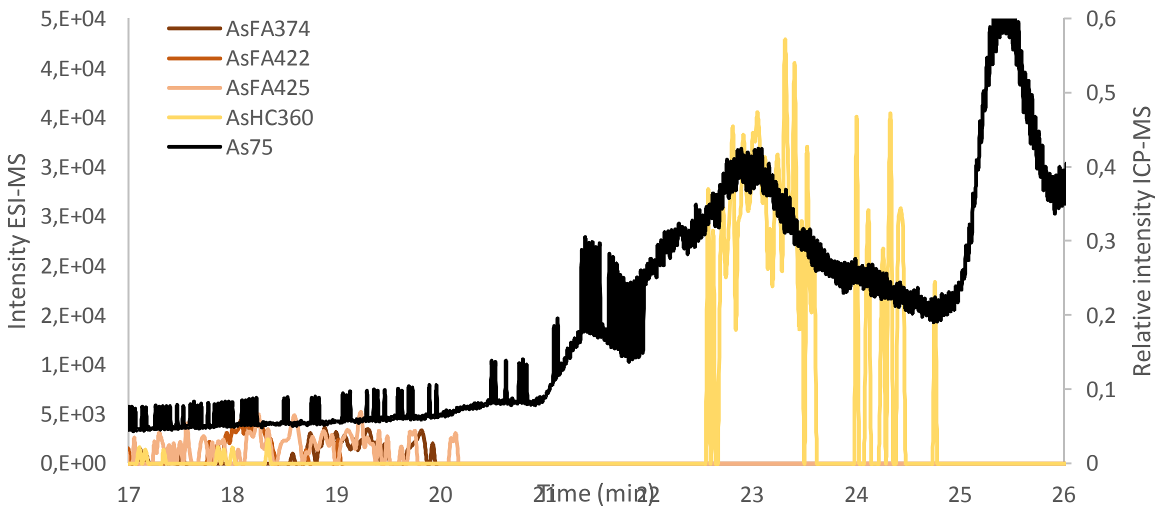

a) AsFAs and AsHC in AE

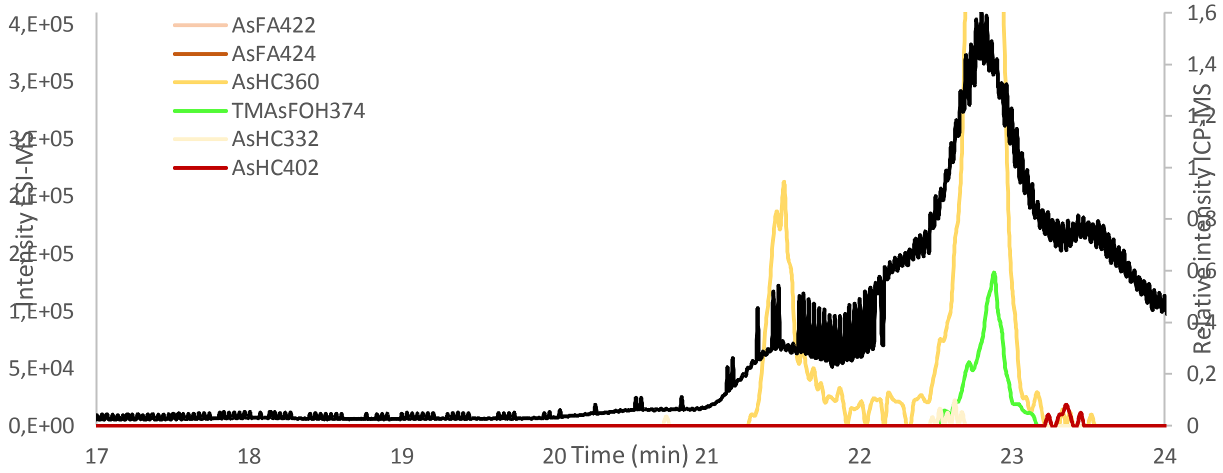

b) AsFAs and AsHC in SL

Fig. S4 Minor traces

a)

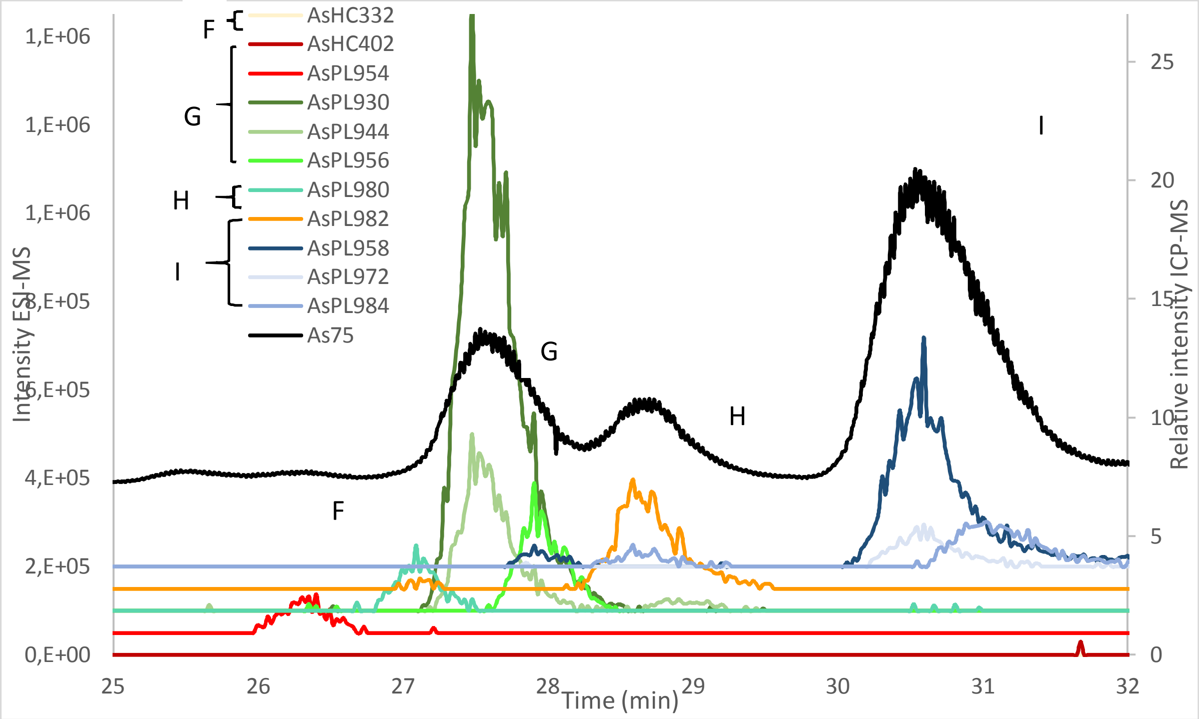

1,E+06
1,E+06
1,E+06
8,E+05
6,E+05
4,E+05
2,E+05
0,E+00
Intensity ESI-MS
Relative intensity ICP-MS
25
20
15
10
5
0
25
26
27
28
29
30
31
32
Time (min)
F
G
H
I
AsHC332
AsHC402
AsPL954
AsPL930
AsPL944
AsPL956
AsPL980
AsPL982
AsPL958
AsPL972
AsPL984
As75

b)

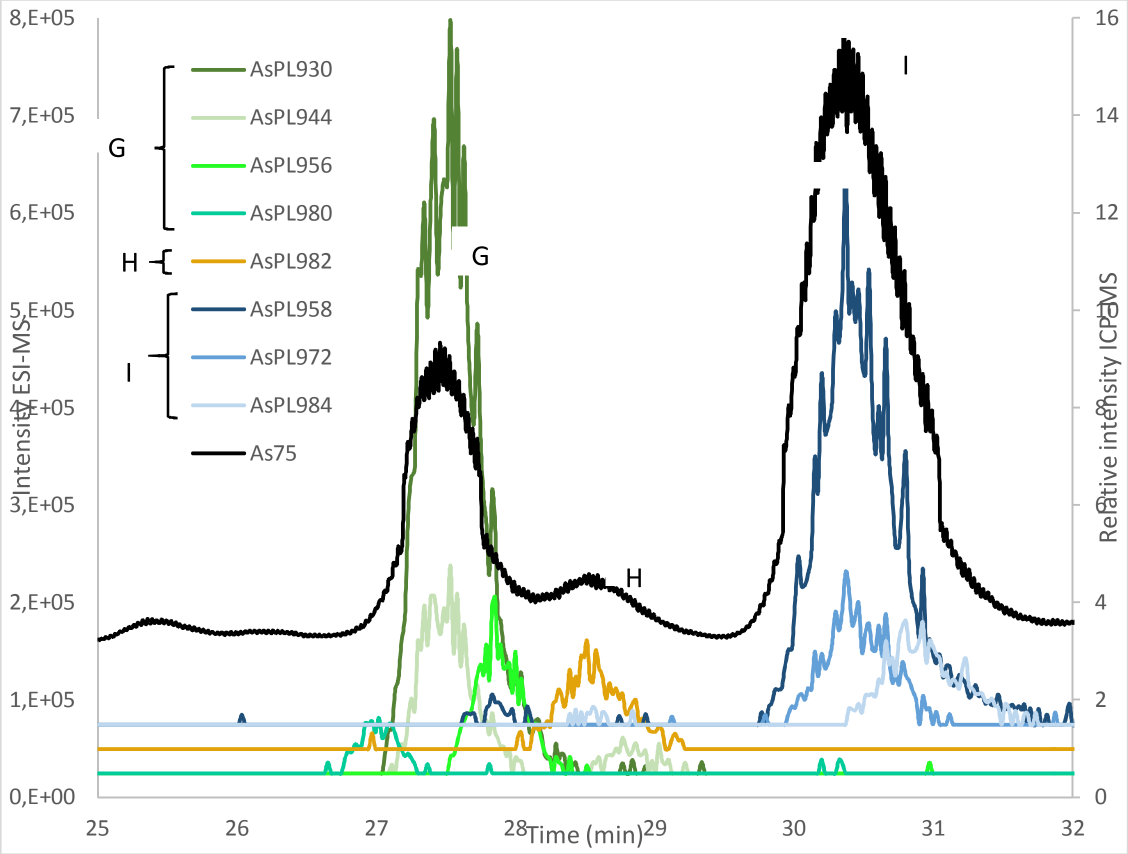

8,E+05
7,E+05
6,E+05
5,E+05
4,E+05
3,E+05
2,E+05
1,E+05
0,E+00
Intensity ESI-MS
Relative intensity ICP-MS
16
14
12
4
2
0
25
26
27
28
29
30
31
32
Time (min)
G
H
I
AsPL930
AsPL944
AsPL956
AsPL980
AsPL982
AsPL958
AsPL972
AsPL984
As75

c)

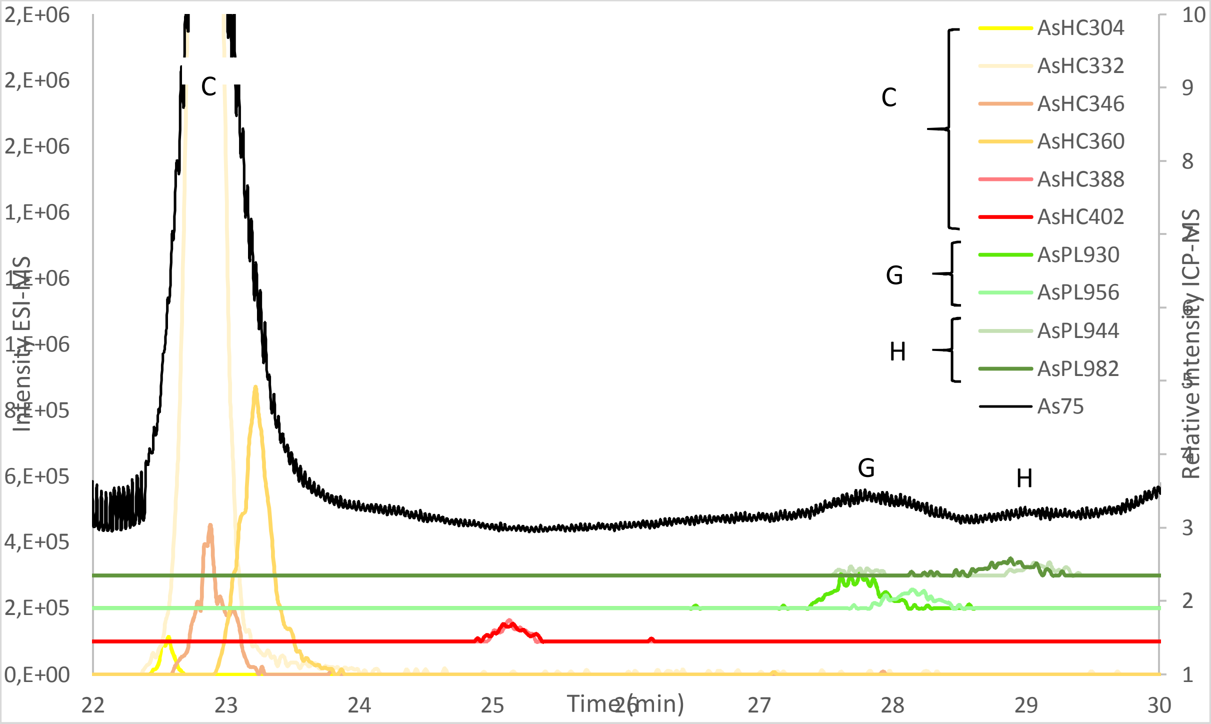

Fig. S5 Overlay of ESI-MS data (coloured lines) and ICP-MS (black, m/z 75). Magnification of close-eluting peaks distinguished with ESI-MS. a) Saccharina latissima young frond b) Alaria esculenta midrib c) Hijiki CRM 7405a. There was a minor time delay for the ESI-MS data and to compensate this the ICP-MS signal was shifted to a 0.7-0.9 minute earlier retention time for all chromatographs

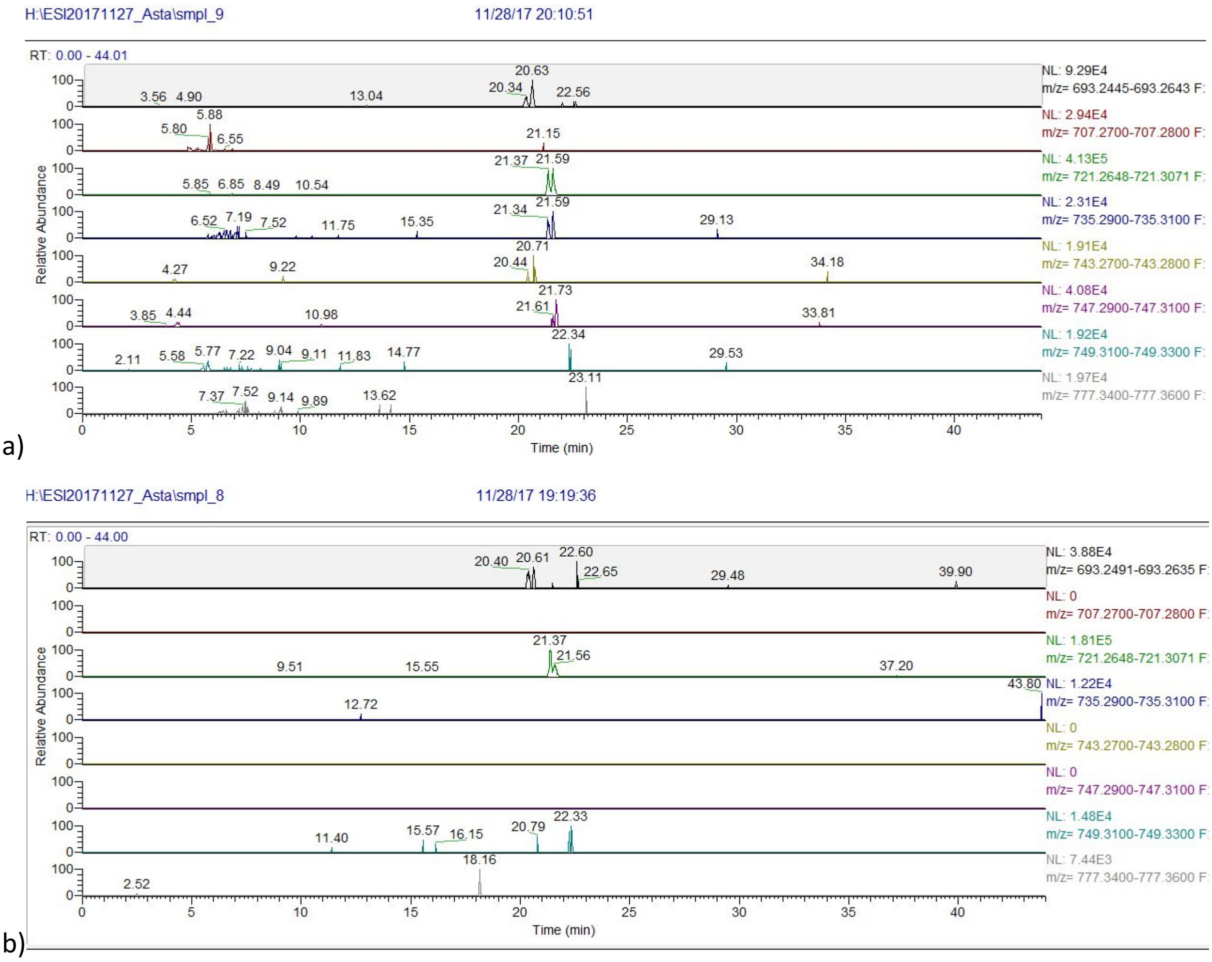

Fig. S6 Excalibur search for previously identified mono acyl AsPLs in S.latissima a) sori b) young frond. Table S2 shows Δm/z for the identified species

MSMS data from Excalibur

Most common fragments for the AsLs were 391 and 409 (as described in Raab et al [3]).

Table S3 Determined MSMS of the samples

short name	m+h	MH+	Δm/z (ppm)	determined mass	short name	m+h	MH+	Δm/z (ppm)	determined mass
AESP	931	C10 H23 O10 As P	-0.614	409.02368	SLYF	931	C10 H23 O10 As P	-2.326	409.02298
	959	C10 H23 O10 As P	*-16.8*	409.01706		959	C10 H23 O10 As P	3.787	409.02548
		C17 H16 O6 As	*-28.5*	391.00461		987	C10 H23 O10 As P	-14.574	409.01797
AES	931	C10 H23 O10 As P	0.731	409.02423	SLSP	931	C10 H23 O10 As P	2.906	409.02512
		C17 H16 O6 As	-6.614	391.01315			C17 H16 O6 As	-1.448	391.01517
AEHF	931	C10 H23 O10 As P	-1.739	409.02322		959	C10 H23 O10 As P	-1.885	409.02316
AEF	959	C10 H23 O10 As P	3.42	409.02533		987	C10 H23 O10 As	-13.82	409.0183
AEM	931	C10 H23 O10 As P	-1.959	409.02313	SLHF	931	C10 H23 O10 As P	-3.377	409.02255
	959	C10 H23 O10 As P	-0.687	409.02365		957	C10 H23 O10 As P	0.877	409.02429
AEM	931	C10 H23 O10 As P	-1.054	409.0235		987	C10 H23 O10 As P	2.149	409.02481
		C17 H16 O6 As	-1.371	391.0152		1001	C11 H25 O10 As P	-0.216	**423.03949**
	959	C10 H23 O10 As P	-2.692	409.02283		1015	C10 H23 O10 As P	-16.066	409.01736
	987	C10 H23 O10 As P	-9.709	409.01996	SLS	931	C10 H23 O10 As P	-3.744	409.0224
Hijiki	959	C10 H23 O10 As P	-1.128	409.02347	SLOF	931	C10 H23 O10 As P	-2.105	409.02307
	961	C10 H23 O10 As P	-1.739	409.02322		959	C10 H23 O10 As P	3.347	409.0253
	987	C10 H23 O10 As P	-0.614	409.02368		987	C10 H23 O10 As P	-1.348	409.02338
		C2 H8 O As	3.473	122.97899		1001	C11 H25 O10 As P	-1.303	**423.03903**
	333	C2 H4 As	-2.119	102.95213		1015	C10 H23 O10 As P	0.437	409.02411
		C17 H38 O As	4.22	333.21472	SLOF	931	C10 H23 O10 As P	-0.467	409.02374
Hijiki	333	C17 H38 O As	-2.382	333.21252			C17 H16 O6 As	-3.008	391.01456
		C2 H8 O As	-1.893	122.97833		959	C10 H23 O10 As P	-3.964	409.02231
		C2 H6 As	-4.841	104.96749		987	C11 H25 O10 As P	-11.964	423.03452
		C2 H4 As	-6.49	102.95168		1001	C18 H21 O5 As P	-0.681	423.03342
	959	C10 H23 O10 As P	-2.105	409.02307	SLOF	931	C10 H23 O10 As P	-3.744	409.0224
		C10 H21 O9 As P	1.006	391.01376		959	C10 H23 O10 As P	0.657	409.0242
	961	C10 H23 O10 As P	0.804	409.02426		987	C10 H23 O10 As P	0.951	409.02432
	987	C10 H23 O10 As P	-4.795	409.02197		1001	C11 H25 O10 As P	-0.854	423.03922

AE= Alaria esculenta, SL = S. latissima. S=stipe. SP=sporophyll, sori. HF=Holdfast. M=midrib (new stipe). F=Frond. YF= young frond. OF= old frond.

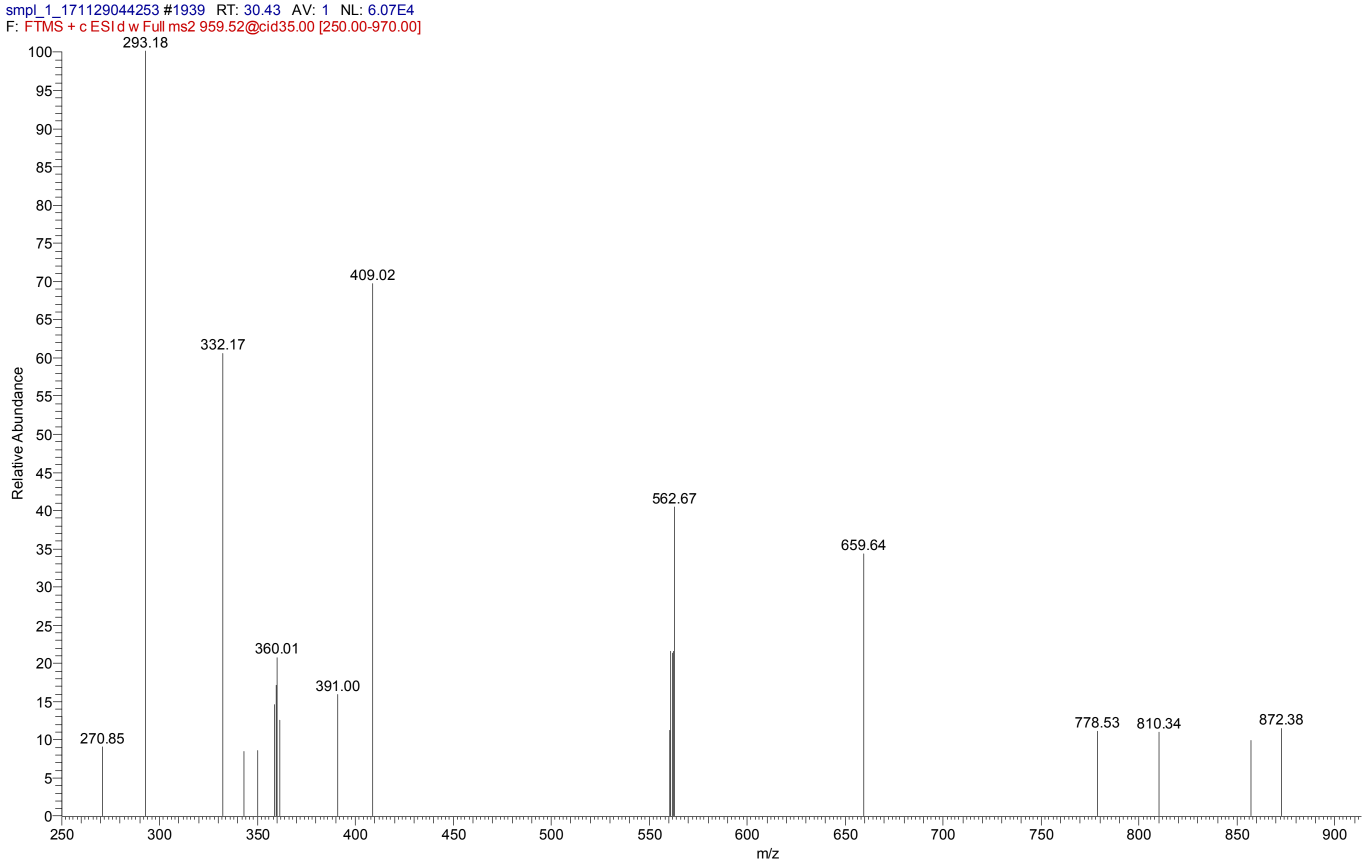
smpl_1_171129044253 #1939 RT: 30.43 AV: 1 NL: 6.07E4
F: FTMS + c ESI d w Full ms2 959.52@cid35.00 [250.00-970.00]
Relative Abundance
m/z
293.18
409.02
332.17
562.67
659.64
360.01
391.00
270.85
778.53
810.34
872.38

a

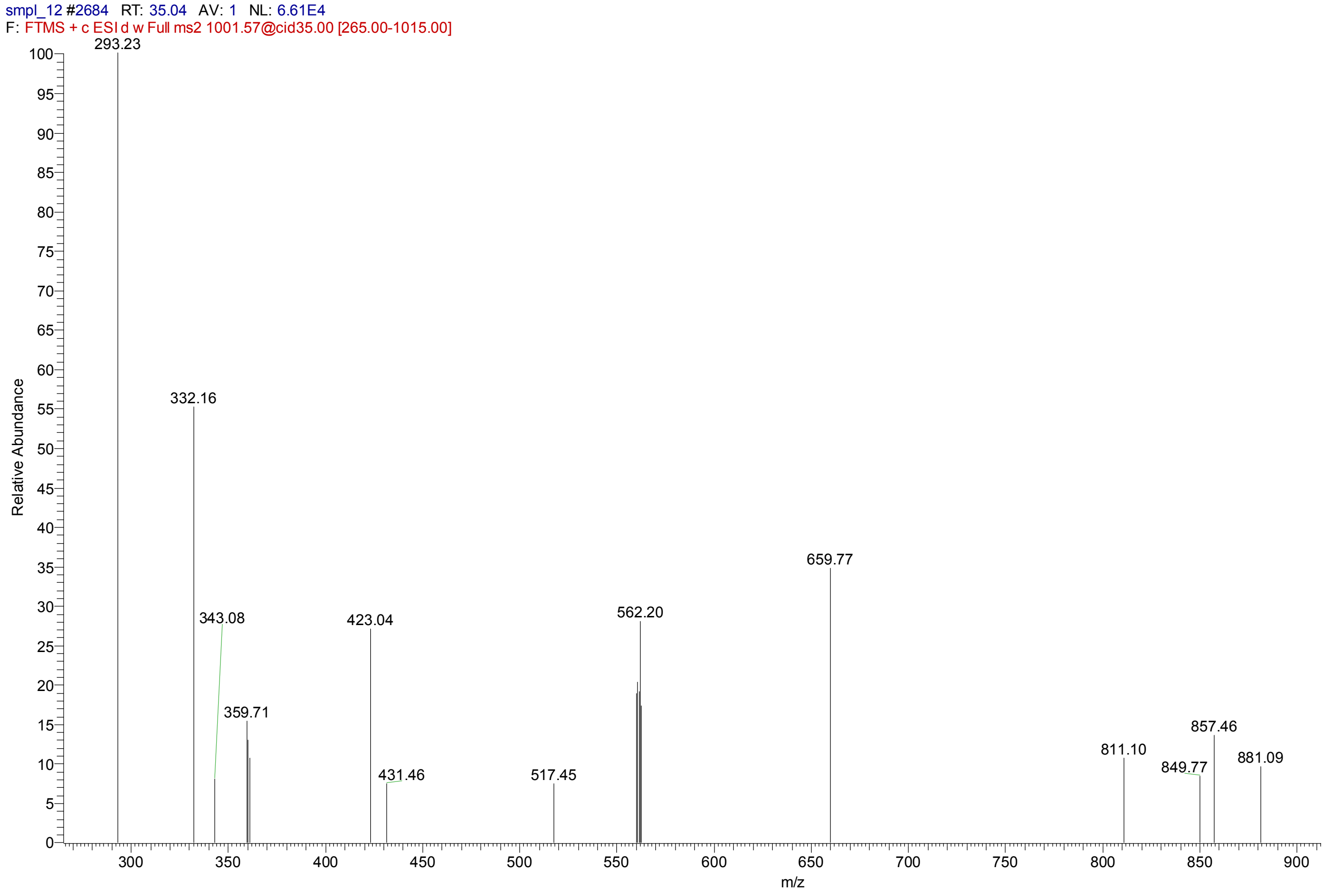
smpl_12 #2684 RT: 35.04 AV: 1 NL: 6.61E4
F: FTMS + c ESI d w Full ms2 1001.57@cid35.00 [265.00-1015.00]
Relative Abundance
m/z
293.23
332.16
659.77
343.08
423.04
562.20
359.71
431.46
517.45
811.10
857.46
849.77
881.09

b

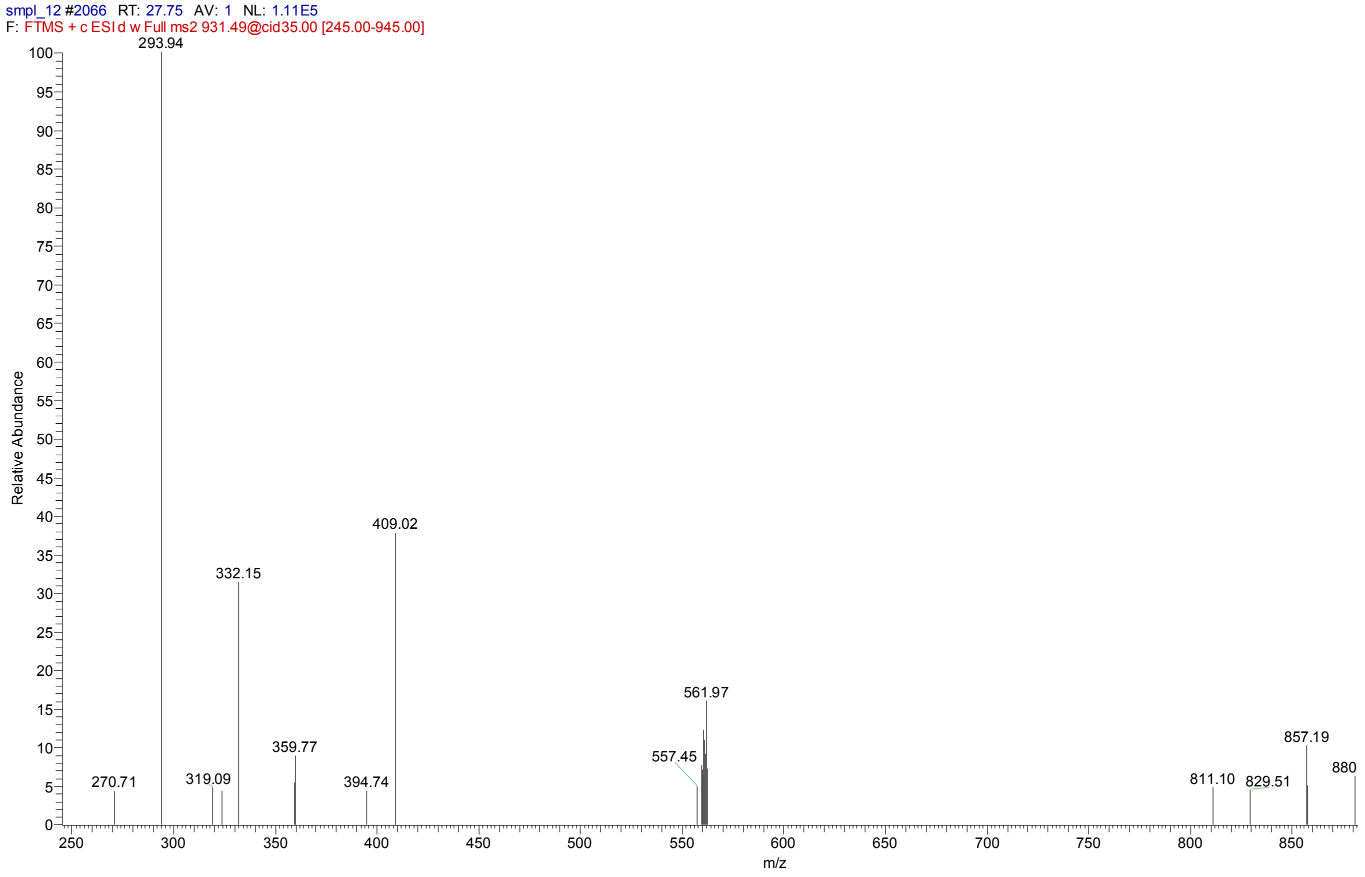
smpl_12 #2066 RT: 27.75 AV: 1 NL: 1.11E5
F: FTMS + c ESI d w Full ms2 931.49@cid35.00 [245.00-945.00]
Relative Abundance
m/z
293.94
409.02
332.15
561.97
359.77
557.45
857.19
880
270.71
319.09
394.74
811.10
829.51

c

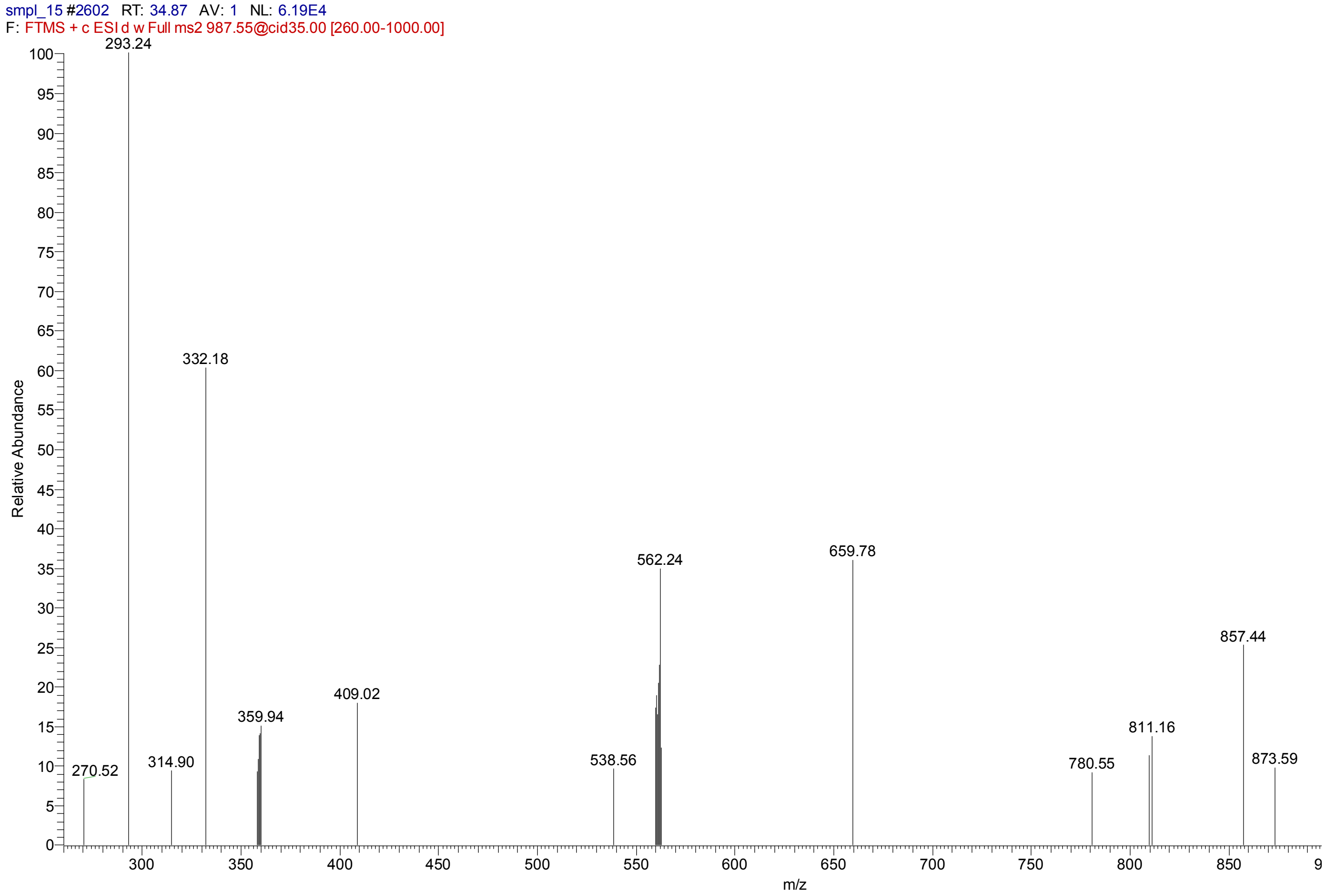
smpl_15 #2602 RT: 34.87 AV: 1 NL: 6.19E4
F: FTMS + c ESI d w Full ms2 987.55@cid35.00 [260.00-1000.00]
Relative Abundance
m/z
293.24
332.18
659.78
562.24
857.44
409.02
359.94
811.16
314.90
538.56
780.55
873.59
270.52

d

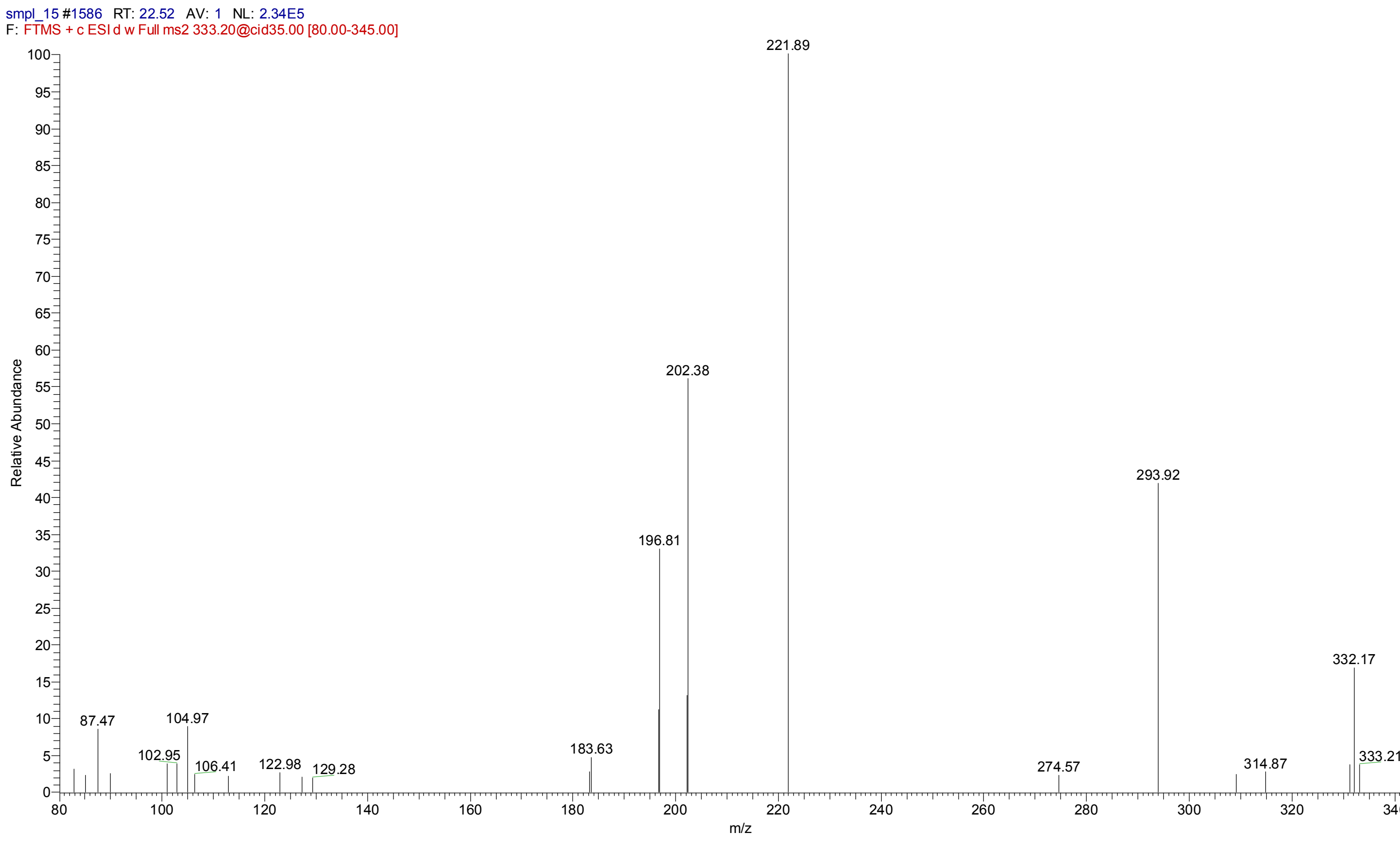
smpl_15 #1586 RT: 22.52 AV: 1 NL: 2.34E5
F: FTMS + c ESI d w Full ms2 333.20@cid35.00 [80.00-345.00]
221.89
202.38
196.81
293.92
332.17
87.47
104.97
102.95
106.41
122.98
129.28
183.63
274.57
314.87
333.21
Relative Abundance
m/z

e

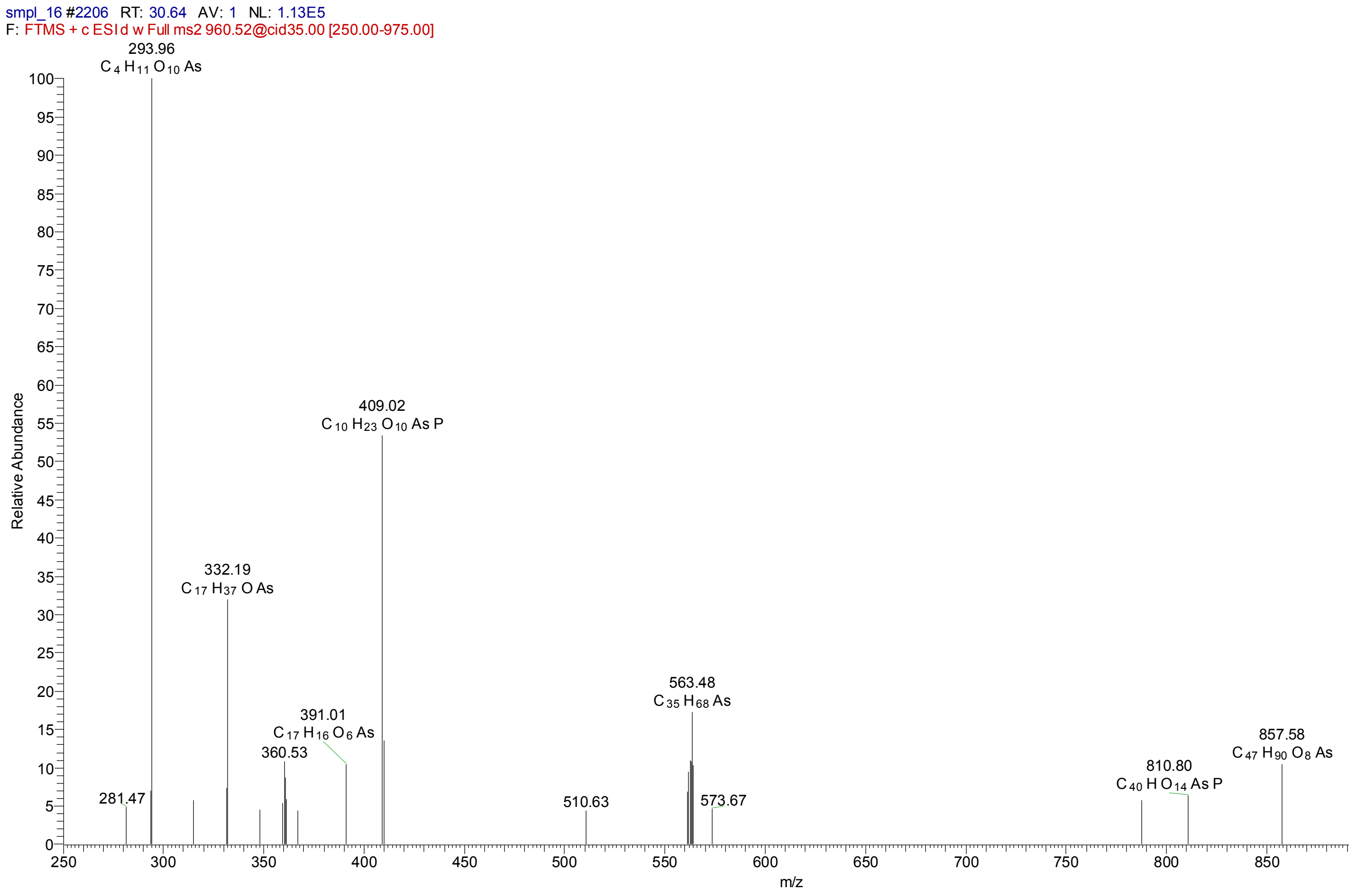
smpl_16 #2206 RT: 30.64 AV: 1 NL: 1.13E5
F: FTMS + c ESI d w Full ms2 960.52@cid35.00 [250.00-975.00]
293.96
C 4 H 11 O 10 As
409.02
C 10 H 23 O 10 As P
332.19
C 17 H 37 O As
391.01
C 17 H 16 O 6 As
360.53
563.48
C 35 H 68 As
857.58
C 47 H 90 O 8 As
810.80
C 40 H O 14 As P
281.47
510.63
573.67
Relative Abundance
m/z

f

g

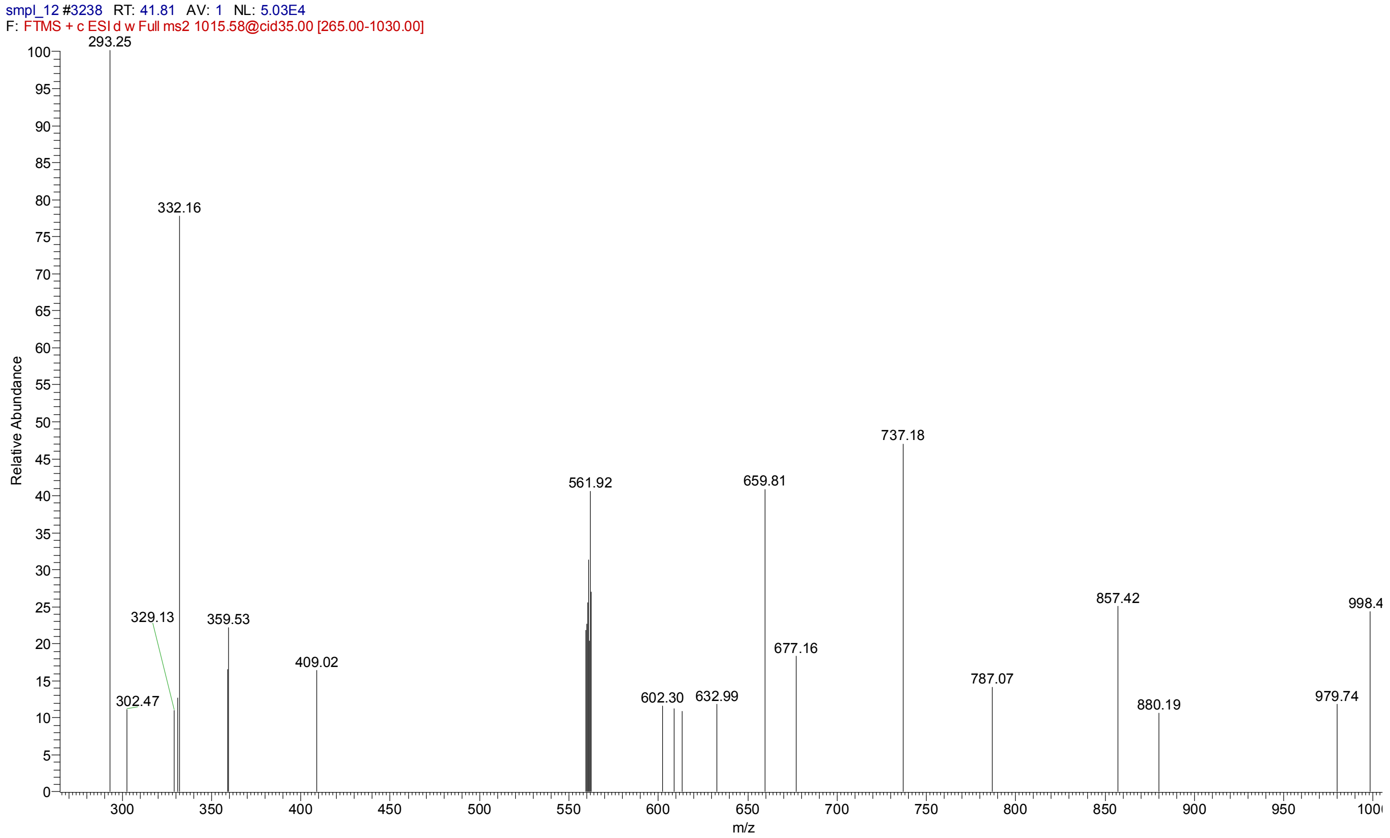

h

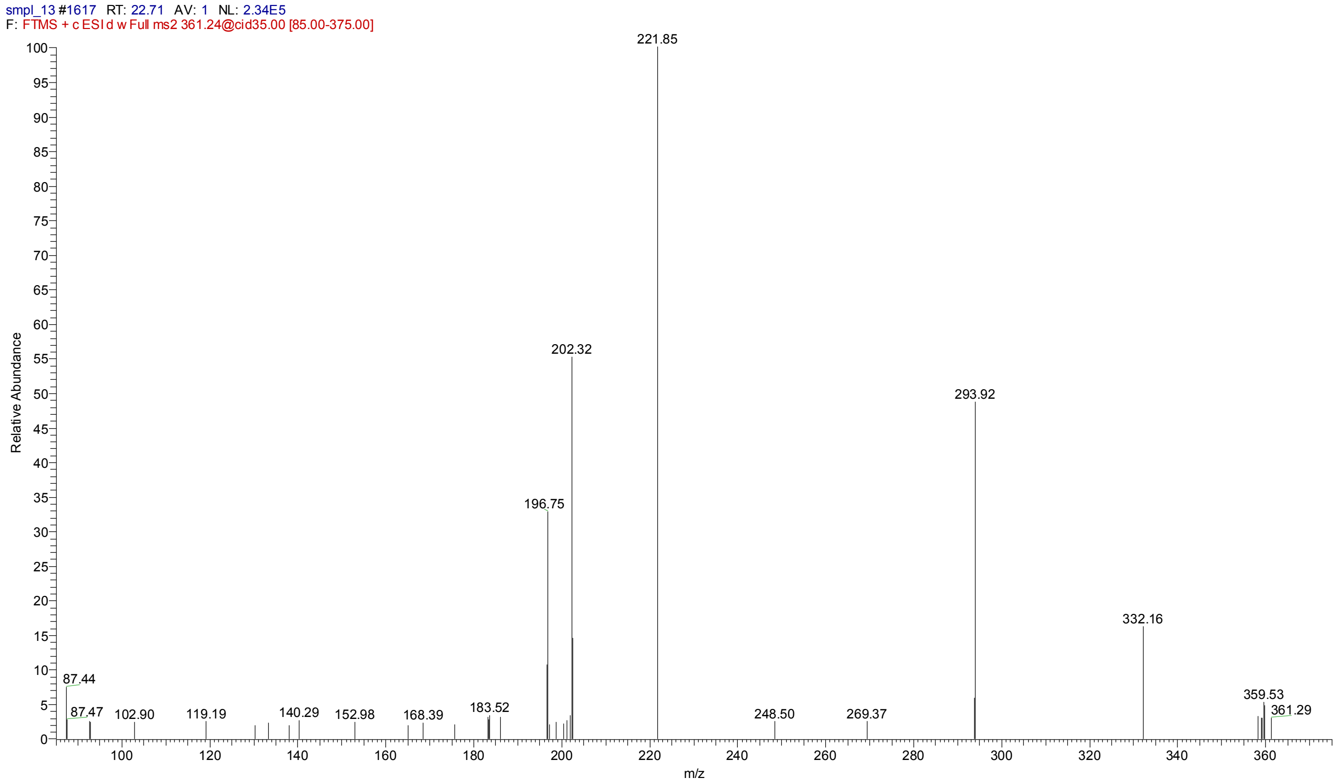

Fig. S7 Example MSMS mass spectras for each species with MSMS. A) Smpl1 (959) – Alaria esculenta sporophyll , b) Smpl12 (1001) – Saccharina latissima old frond c) smpl12 (931), S. latissima old frond, d) Smpl15 (987) – Hijiki, e) smpl15 (333) Hijiki f) Smpl16 (961) Hijiki g) Smpl12 (1015) S. latissima old frond h) Smpl14 (361) S. latissima Old frond

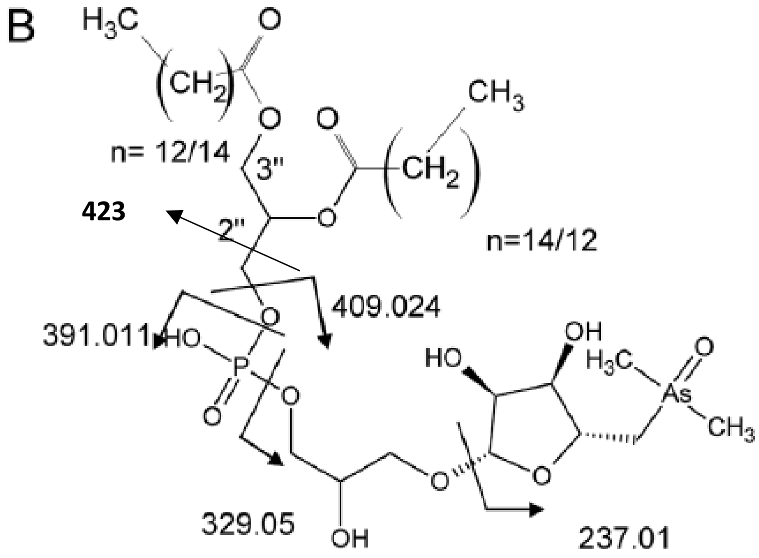

Fig. S8 Figure adapted from Raab et al [3]. Fragments found here are similar, where the fragment with m/z 423 fits the formula and pattern indicated here in red

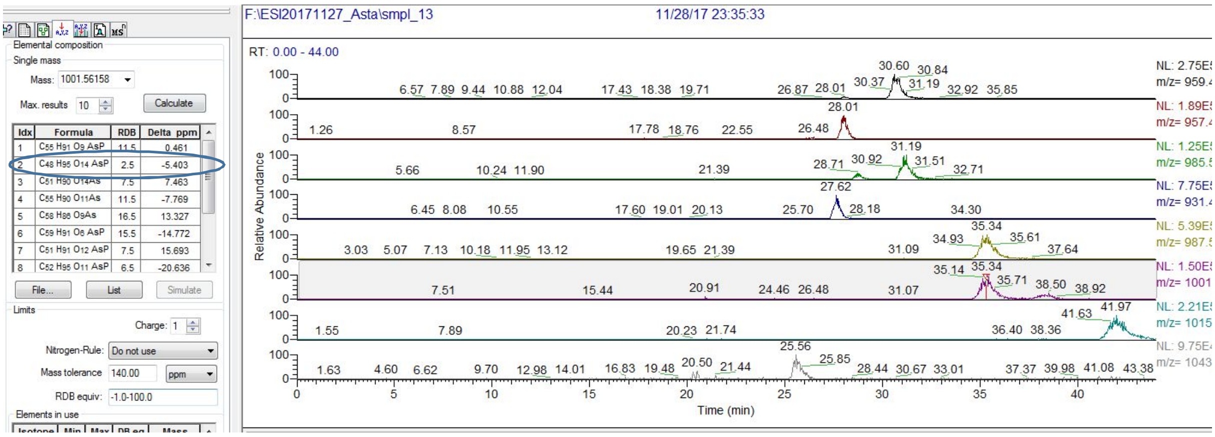

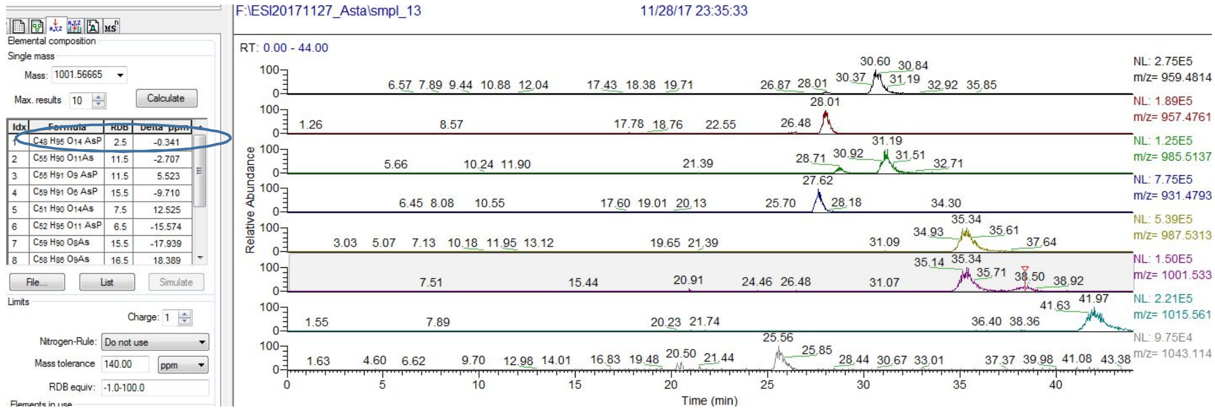

Instability of AsPLs?

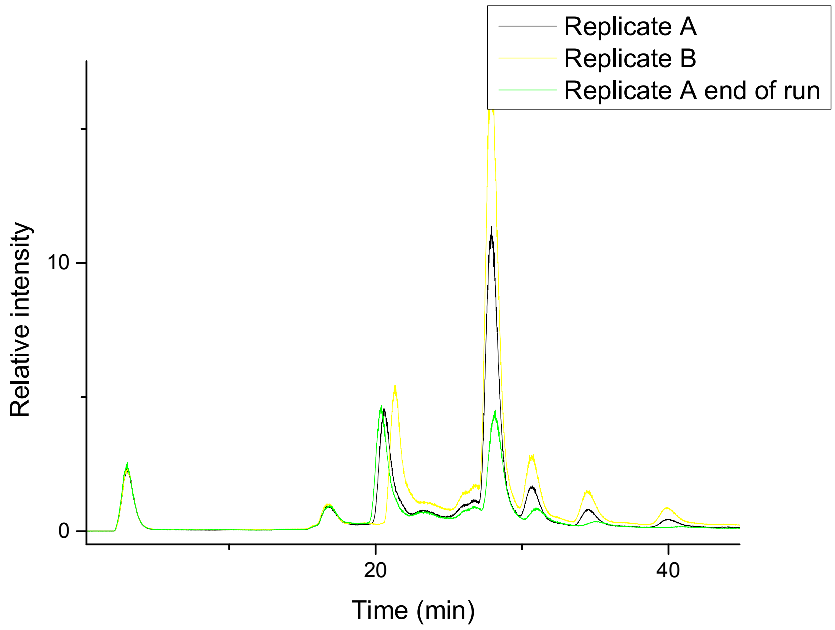

a)

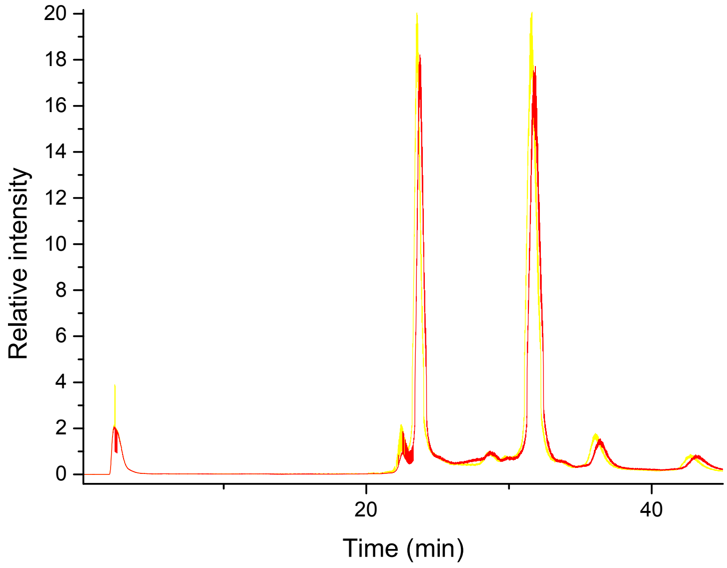

b)

Fig. S9 a) Hijiki batch A b) Hijiki batch B

Replicates are similar for both batches (sample weighed into two separate vials and extracted). For the first batch, 1 replicate was re-analysed at the end of the run (approx. 22+ hours later), and it can be seen that the intensity is lower for AsPLs than it was before. The AsHCs and void peak are the same. It may indicate that the AsPLs may be rather unstable, which must be investigated in greater detail. However, since the peak at the void volume does not increase it is uncertain what has happened to the arsenic. For batch B, the Hijiki was only analysed at the end of the run, and the column recovery was acceptable.

Comparison of the two extractions of the (same) samples

Some problems arose in Abdn with the measurement. There is a problem with automatic PA tuning when running in oxygen mode, **Fig. S*11***b. By identifying the jump spot the data can be manually corrected, **Fig. S*11***c.

Alaria esculenta

The first few runs were under different HPLC settings. This resulted in a different elution for some *A. esculenta* samples. Hence, there were minor issues with quantification for the samples analysed in Abdn. Despite this, the data shows reasonably good comparison in the "pattern", although higher concentrations of AsLs are found with the second batch (as described in manuscript.

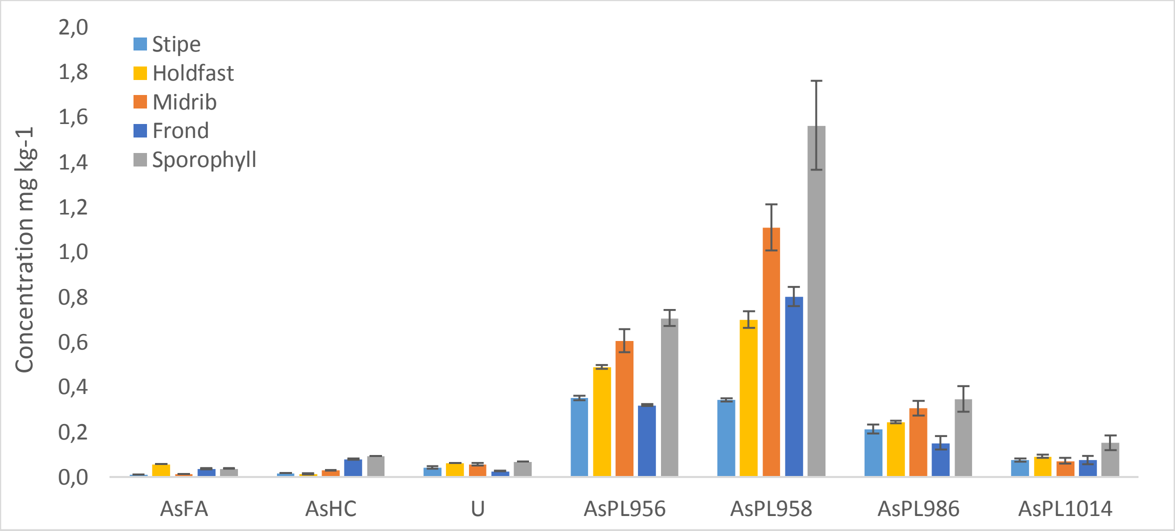

a)

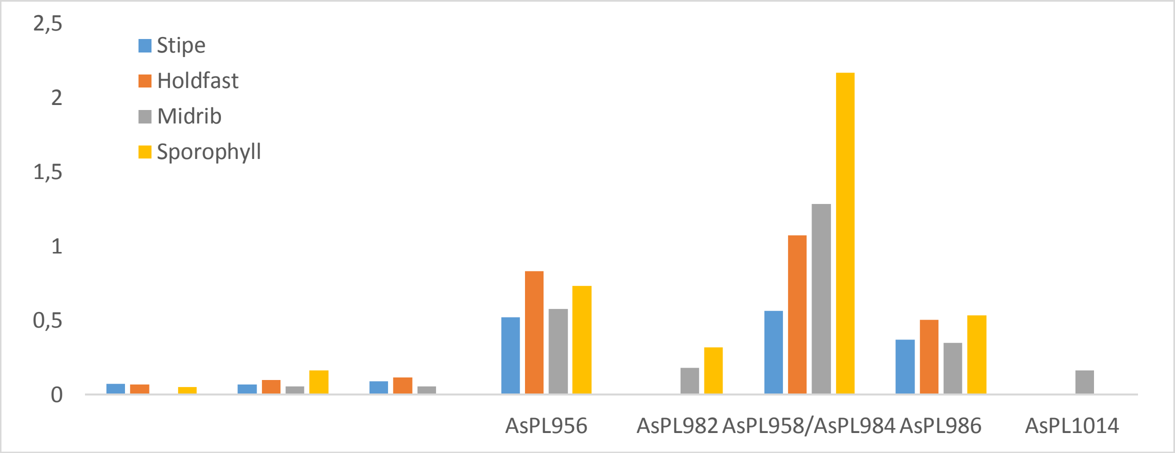

b)

Fig. S10 Samples of Alaria esculenta. A) Matís B) Abdn

Problems with *A. esculenta* samples at Abdn. Not analysed all with the same HPLC method, retention times very different. Graph strange for the frond (and excluded in quantification):

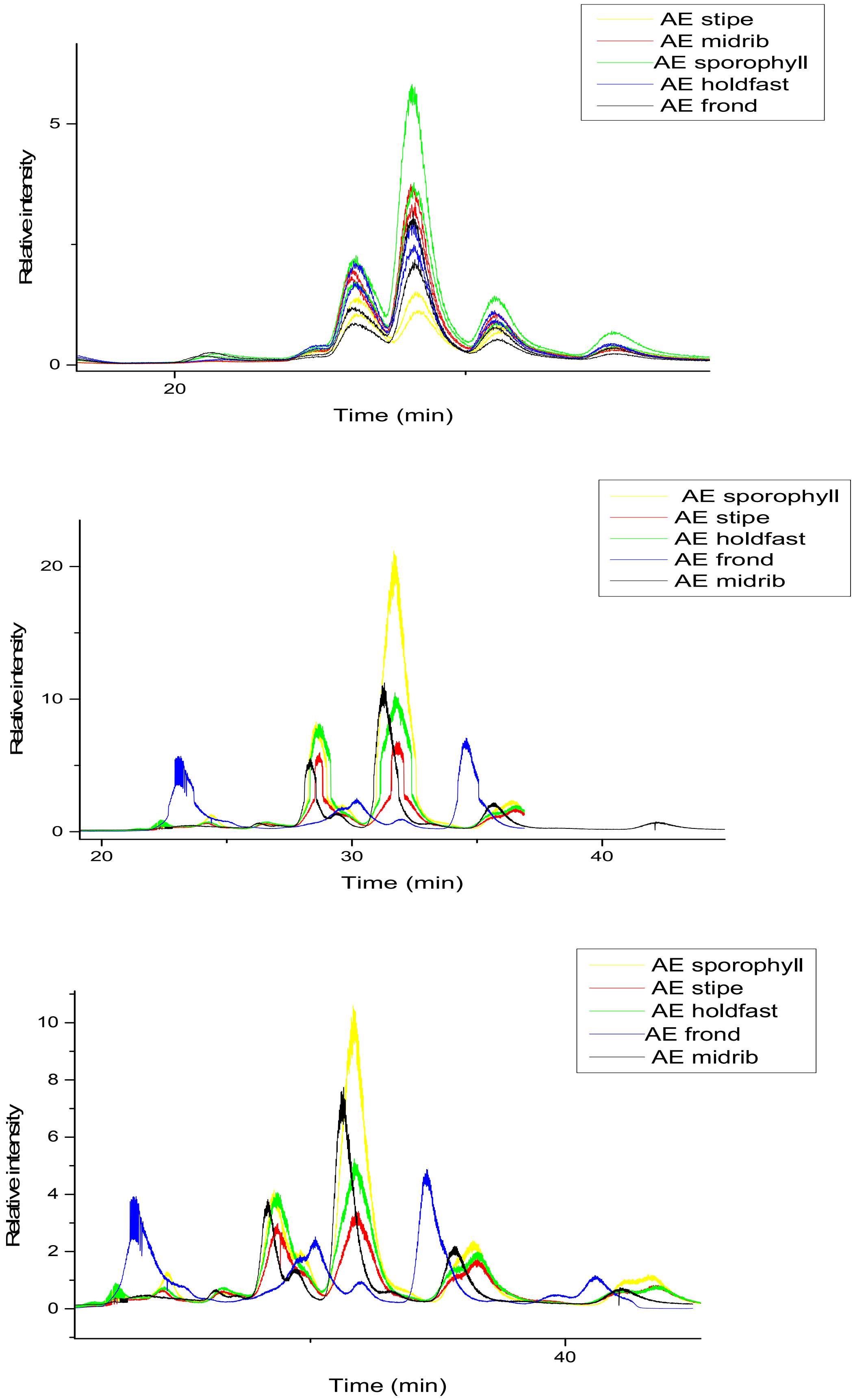

Fig. S11 Samples of Alaria esculenta (AE) measured on HPLC-ICPMS A) Matís B) Abdn – prior to manual PA correction c) Abdn after manual PA correction

Saccharina latissima

There was excellent reproducability between the two batches. The increasing “trend“ was seen again for the lighter AsPLs although it was a bit different for the heavier ones, **Fig.** ***S12***. May that be due to quantification differences or potentially that there may be some instability in the dry seaweed for heavier AsPLs.

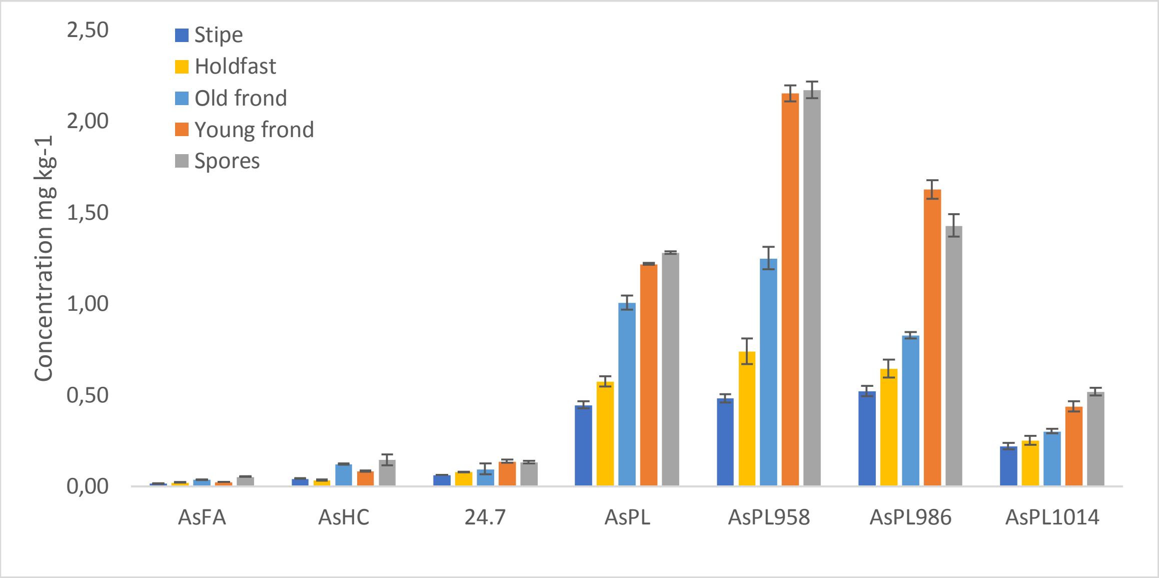

a)

b)

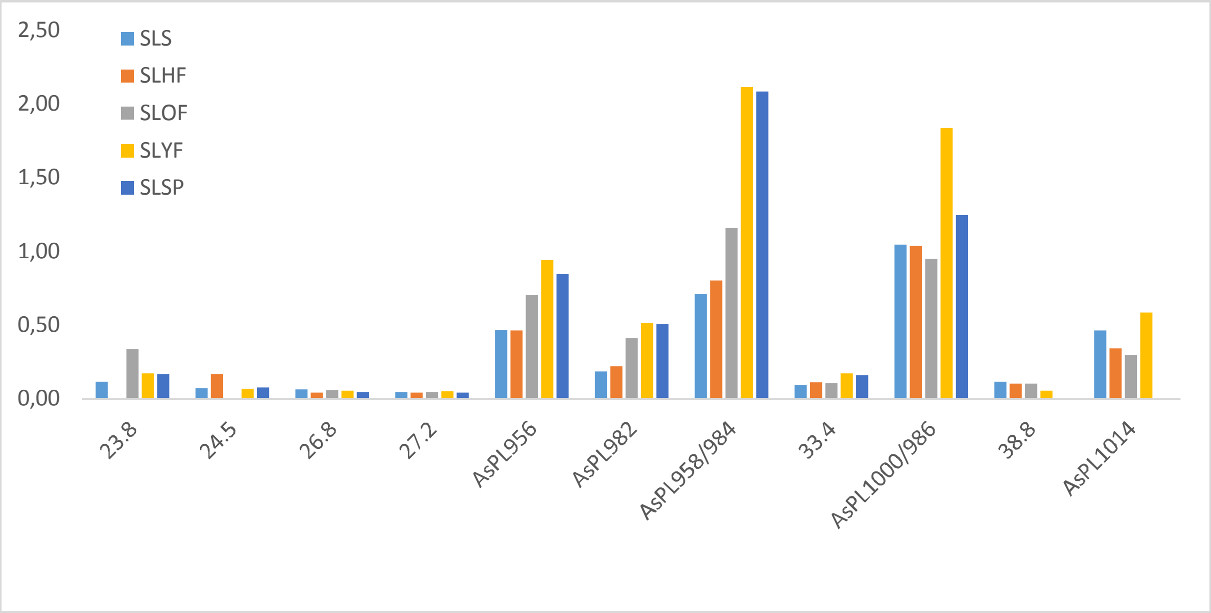

Fig. S12 Samples of Saccharina latissima A) Matís B) Abdn

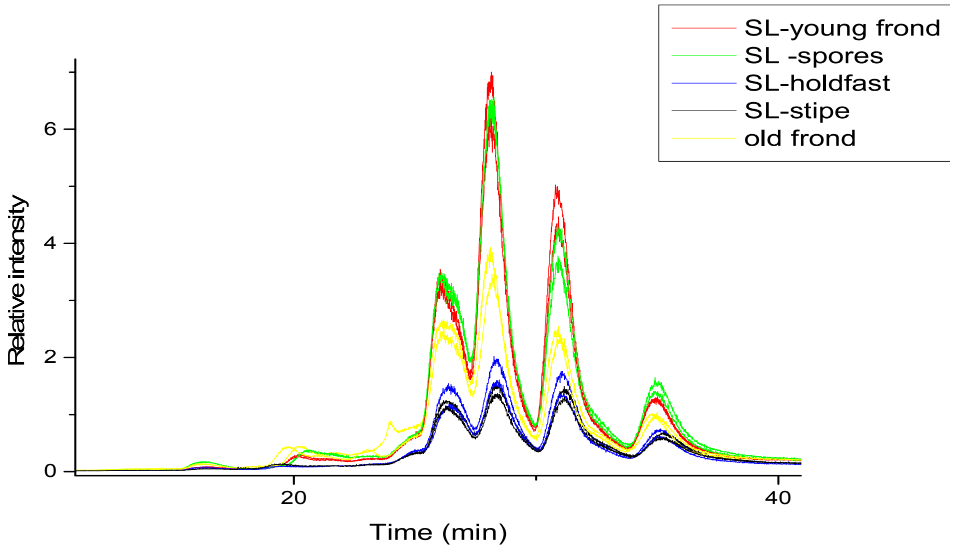

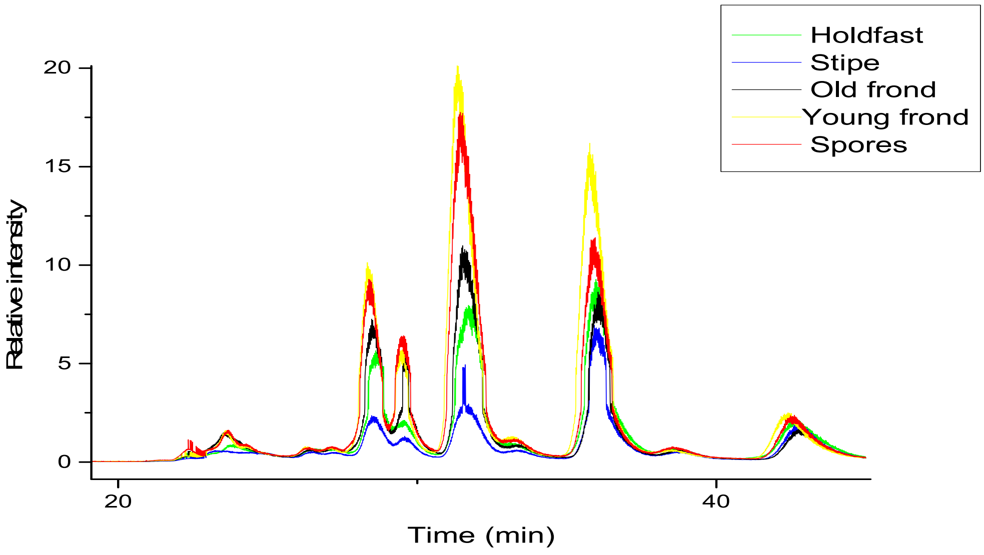

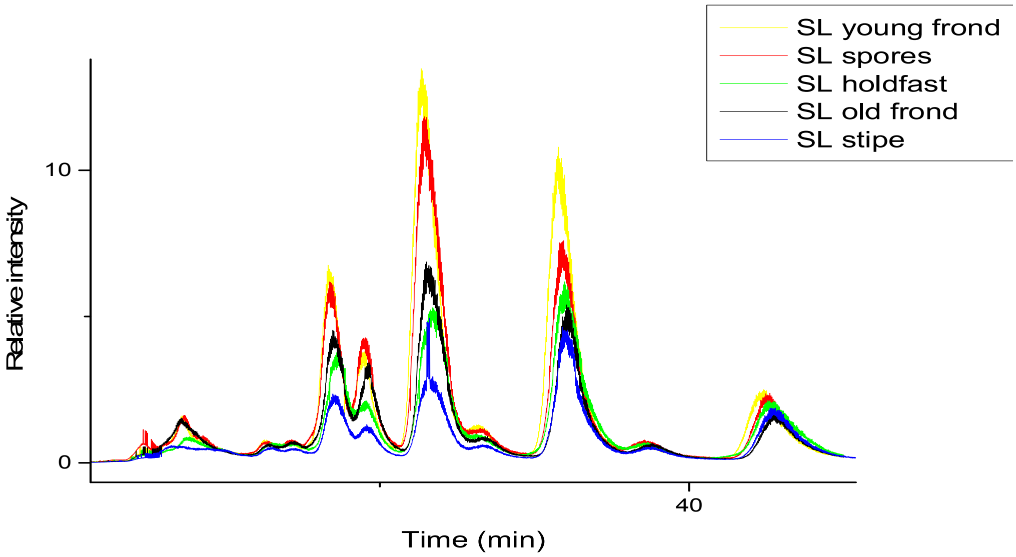

Fig. S13 Samples of Saccharina latissima A) Matís B) Abdn, prior to manual PA correction c) after PA correction

Arsenosugars

Table S4 Quantification of water soluble arsenic in S. latissima

	**Stipe**	Holdfast	Old frond	Young frond	Sori
AsSugOH	1.9 ± 0.2	2.59 ± 0.05	2.9 ± 0.1	3.6 ± 0.6	2.7 ±0.8
DMA	0.09 ± 0.02	0.35 ± 0.01	0.35 ± 0.02	0.34 ± 0.04	0.29 ± 0.04
AsSugPO4	6.8 ± 1.1	13.2 ± 0.2	12.1 ± 0.9	10.1 ± 3.3	8.9 ± 1.2
AsSugSO3	18.2 ± 2.6	74.2 ± 1.0	60.7 ± 5.2	72.2 ± 9.8	53.9 ± 7.2
U			0.5 ± 0.07	0.7 ± 0.1	0.61 ± 0.04
AsSugSO4			0.37 ± 0.02	0.35 ± 0.18	0.30 ± 0.03

Table S5 Quantification of water soluble arsenic in A. esculenta

	Stipe	Holdfast	Midrib	Frond	Sporophyll
AsSugOH	0.28 ± 0.06	1.2 ± 0.05	1.3 ± 0.1	4.8 ± 1.0	1.48 ± 0.03
DMA	0.05 ± 0.07	0.14 ± 0.01		0.22 ± 0.02	0.05 ± 0.01
AsSugPO4	7.5 ± 0.8	9.7 ± 0.5	3.2 ± 0.1	7.1 ± 0.3	11.8 ± 0.1
AsSugSO3	19.7 ± 1.5	47.3 ± 2.3	18.7 ± 1.1	48.5 ± 0.6	46.6 ± 1.1
U		0.15 ± 0.07		0.38 ± 0.02	0.23 ± 0.04
AsSugSO4	0.08 ± 0.01	0.17 ± 0.03	0.01 ± 0.002	0.31 ± 0.01	0.11 ± 0.003

For S.latissima and A.esculenta As(III) would elute with AsSugarOH and As(V) with AsSugarSO4 (as tested by spiking). Low quantities of iAs are expected.

Table S6 Quantification of water soluble arsenic in Hijiki (n=3) in mg kg^{-1}

	Hijiki	Reported in Wolle et al [4]
AsSugOH+AsIII	0.42 ± 0.05	0.386 ± 0.014
DMA	0.62 ± 0.06	0.468 ± 0.023
AsSugPO4	1.5 ± 0.1	1.113 ± 0.029
AsSugSO3	1.1 ± 0.1	0.546 ± 0.041
AsSugSO4 + AsV	14.6 ± 1.2	12.3 ± 0.1

References

[1] R.A. Glabonjat, G. Raber, K.B. Jensen, J. Ehgartner, K.A. Francesconi, Quantification of Arsenolipids in the Certified Reference Material NMIJ 7405-a (Hijiki) using HPLC/Mass Spectrometry after Chemical Derivatization, Anal Chem, 86 (2014) 10282-10287.
[2] A.H. Petursdottir, J. Rodrigues de Jesus, H. Gunnlaugsdottir, J. Feldmann, Quantification of labile and stable non-polar arsenolipids in commercial fish meals and edible seaweed samples, Journal of Analytical Atomic Spectrometry, 33 (2018) 102-110.
[3] A. Raab, C. Newcombe, D. Pitton, R. Ebel, J. Feldmann, Comprehensive Analysis of Lipophilic Arsenic Species in a Brown Alga (Saccharina latissima), Analytical Chemistry, 85 (2013) 2817-2824.
[4] M.M. Wolle, S.D. Conklin, Speciation analysis of arsenic in seafood and seaweed: Part II-single laboratory validation of method, Anal Bioanal Chem, 410 (2018) 5689-5702.
